# Black nurses in the nursing profession in Canada: a scoping review

**DOI:** 10.1186/s12939-022-01673-w

**Published:** 2022-07-23

**Authors:** Keisha Jefferies, Chelsa States, Vanessa MacLennan, Melissa Helwig, Jacqueline Gahagan, Wanda Thomas Bernard, Marilyn Macdonald, Gail Tomblin Murphy, Ruth Martin-Misener

**Affiliations:** 1 School of Nursing, Faculty of Health, Dalhousie University, Halifax, NS Canada; 2 W.K. Kellogg Health Sciences Library, Dalhousie University, Halifax, NS Canada; 3 Mount Saint Vincent University, Halifax, NS Canada; 4 Professor Emerita at Dalhousie University, Halifax, NS Canada; 5 Research and Innovation, Nova Scotia Health, Halifax, NS Canada

**Keywords:** African Descent, Black, Nursing, Racism, Canada

## Abstract

**Background:**

With migration occurring over a series of centuries, dating back to the 1600’s, the circumstance regarding Black people in Canada is a complex account. A plethora of social issues and the failure to adequately acknowledge and reconcile historical issues, has resulted in health inequity, disparities and knowledge gaps, related to the Black population in Canada. In nursing, historical records indicate a legacy of discrimination that continues to impact Black nurses. The profession has begun reckoning with anti-Black racism and the residual effects. This scoping review sought to chart the existing evidence on Black nurses in the nursing profession in Canada.

**Methods:**

JBI methodology was used to search peer-reviewed evidence and unpublished gray literature. Sources were considered for inclusion based on criteria outlined in an a priori protocol focusing on: 1) Canada 2) Black nurses and 3) nursing practice. No restrictions were placed on date of publication and language was limited to English and French. All screening and extractions were completed by two independent reviewers.

**Results:**

The database search yielded 688 records. After removing duplicates, 600 titles and abstracts were screened for eligibility and 127 advanced to full-text screening. Eighty-two full-text articles were excluded, for a total of 44 sources meeting the inclusion criteria. Seven sources were identified through gray literature search. Subsequently, 31 sources underwent data extraction. Of the 31 sources, 18 are research (*n* = 18), six are commentaries (*n* = 6); one report (*n* = 1) and six are classified as announcements, memoranda or policy statements (*n* = 6). The review findings are categorized into five conceptual categories: racism (*n* = 12); historical situatedness (*n* = 2); leadership and career progression (*n* = 7); immigration (*n* = 4); and diversity in the workforce (*n* = 4).

**Conclusions:**

This review reveals the interconnectedness of the five conceptual categories. Racism was a prominent issue woven throughout the majority of the sources. Additionally, this review captures how racism is exacerbated by intersectional factors such as gender, class and nationality. The findings herein offer insight regarding anti-Black racism and discrimination in nursing as well as suggestions for future research including the use of diverse methodologies in different jurisdictions across the country. Lastly, the implications extend to the nursing workforce in relation to enhancing diversity and addressing the ongoing nursing shortage.

## Introduction

The health and human rights of people of African descent have been brought to the forefront in the wake of the COVID-19 pandemic, which has exposed the most vulnerable, marginalized and oppressed sectors of society [1]. The televised mistreatment of people of African descent has led to mounting calls for action to end anti-Black racism, particularly through research and the collection of race-disaggregated data [2, 3]. Anti-Black racism is defined as the specific processes, decisions and policies that intentionally or unintentionally discriminate against Black people [4]. The impact of anti-Black racism permeates a multitude of sectors in Canadian society including education, healthcare and nursing specifically. Addressing anti-Black racism and discrimination in the nursing profession warrants research that investigates how social constructs including heteronormativity, gender identity/expression, class and disability interact with race to influence health [5]. As one of the largest and most trusted health care professions, nursing is in an optimal position to address the lingering effects of historic oppression in healthcare and throughout society more broadly. Precisely, promoting diversity and inclusivity within the profession is suggested as one approach to address issues of belonging as well as enhance care delivery for patients [6]. Ergo, in light of the increasing focus on the implications of racism in Canada and globally, it is necessary to begin to chart existing evidence concerning Black nurses to illuminate insight for practice and future research. Finally, the title of this scoping review, and the language used to describe the participants, differs from the published protocol [7]. The published protocol describes the participants as *African Canadian nurses* however, after careful review of the existing and emerging literature in this area, the terminology was modified from *African Canadian nurses* to *Black nurses*. The term *Black*has gained global recognition as a concept that extends beyond biology or genetics to include a more politicized and widely understood meaning that encompasses the historical and social aspects pertaining to people of African descent [8].

### Black nurse trailblazers: a launchpad for black nurses

Understanding the historical context of nursing is an important first step in situating a review regarding Black nurses in the nursing profession in Canada. Historically, in the global west, people of African descent struggled to enter, practice and have their contributions recognized in the nursing profession [9]. For example, Mary Seacole (1850s), a Jamaican-born nurse who was based in London, England provided care to British soldiers during the Crimean War [10]. Seacole, who was as active and innovative as other prominent historical nurse figures, was all but erased from the historical nursing record until recently [10]. Similarly, in the United States, African Americans were banned from entering nursing training facilities until the 1870s, with Mary Mahoney (1879) being the first African American to become a nurse [11, 12].

In Canada, the historical nursing record reveals a similar legacy of segregation and discrimination, which scholars argue remains prevalent today. The first nursing school in Canada opened in 1874, with the first Baccalaureate program offered in 1919 [13, 14]. However, in the female-dominated profession, Black women were not permitted to train as nurses until the late 1940’s [9, 15]. At the time, Black women who aspired to be a nurse, were instead instructed to travel and train in the United States [9, 15]. Notwithstanding, Black women in Canada, who were committed to care provision despite being denied the opportunity to train formally, eventually formed the Black Cross Nurses in Canada [16]. This auxiliary group was established in the 1920s and modelled after the Red Cross. The Black Cross Nurses comprised a network that enabled Black women, who were not formally trained nurses, to provide health-related service to communities [16]. Examples of services provided by the Black Cross nurses included answering sick calls, assisting parents and children as well as domestic duties [16]. In addition to non-licensed care practices provided by Black women, community-level activism was the catalyst that ignited the process of challenging the systemic barriers encountered by Black women entering nursing. Early activism was led in large part by Pearleen Oliver, who despite not being a nurse, had a pivotal role in shifting the overt discriminatory admissions policies in nursing training facilities in Canada [15]. Oliver, in collaboration with community-based groups and organizations, including the Nova Scotia Association for the Advancement of Colored People (NSAACP) and the Canadian Negro Citizenship, publicly challenged the exclusion of Black women from nursing schools, marking a momentous period for Black history and Canadian nursing [9, 15].

### The nursing workforce in Canada

Canada currently recognizes four nursing designations, which include licensed practical nurses (LPNs) [or registered practical nurses (RPNs) in Ontario]; registered nurses (RNs), registered psychiatric nurses and; nurse practitioners (NPs) [17]. Of the 448,044 regulated nurses with an active license in Canada, approximately 130,710 are LPNs, 6,115 are registered psychiatric nurses, 304,558 are RNs and 6,661 are NPs [18]. In terms of demographics, the Canadian Institute for Health Information (CIHI) disaggregates data regarding the nursing workforce according to gender as binary and age [18] however, critical demographic indicators including race/ ethnicity, sexual orientation, gender identity/ expression, class as well as disability are missing. In the absence of these data to advance knowledge and inform policy development in a comprehensive and evidence-based manner, there is a growing call for the collection of race-disaggregated data [2]. The necessity for the collection of race-disaggregated data becomes apparent in considering one of the main crises impacting the nursing workforce in Canada, which is the nursing shortage. The nursing shortage has been described as an ongoing health system issue that has been further exacerbated by multifaceted population health issues, including the COVID-19 pandemic. Strategies to address the shortage include considerations in three critical areas – RN production; RN retention; RN deployment, with emphasis on the in-migration of internationally educated nurses (IENs), attrition in nursing education programs, and workforce productivity [19, 20].

### Considerations in nursing education

Another area of concern that directly impacts the Canadian nursing workforce is nursing education. Nursing education encompasses salient aspects of nursing training including curricula and program admissions. Canadian nursing curricula has been criticized as relying too heavily on a Eurocentric ideological foundation that effectively reinforces prejudice, stereotypes and discrimination towards specific groups [8, 9, 21–25]. Further, nursing curricula has been criticized for failing to incorporate content that would enhance competency related to care delivery for historically marginalized populations [9, 21–25]. Until recently, most nursing curricula omitted content that incorporated the experiences, contributions or basic existence of Black nurses in Canada [8, 9]. To address these gaps in education, researchers and academics are attempting to remedy this problem [8, 21, 23–25]. For example, Blanchet et al. [24] proposed a critical anti-discriminatory pedagogy (CADP) for nursing practice and education. Another salient example of critical shifts in nursing curricula includes the development of a syllabi evaluation tool [23]. Emerging from the need to evaluate and modify the content taught in nursing education, Lane and Waldron [23] created a rubric that evaluates course syllabi. This rubric, which aims to improve nursing curricula through increased representativeness and the elimination of oppressive stereotypes, guides faculty in the development of inclusive and representative syllabi [23].

In terms of program admissions, most institutions do not collect disaggregated data that would offer insight into the representativeness of the student body. However, the underrepresentation of Black students in nursing programs had been argued, in text and opinion sources, as an issue that is exacerbated by institutional policies and implicit bias. Further, the underrepresentation of Black nurses in the profession is suspected as deriving from the underrepresentation of Black students in nursing programs.

### Nursing as a critical practice

Social justice and a critical social approach to health are core values in nursing [24]. However, a shift away from these values has created tension in the profession as nurses struggle to reintroduce these values into nursing practice and education [24]. For example, the issue of diversity in the nursing profession in Canada can be attributed to factors such as stereotypes and discrimination, institutional and systemic barriers (i.e.: financial), as well as a lack of representative mentors and role models [26]. Etowa et al. [27] suggest that in order to increase diversity within the nursing profession in Canada, healthcare organizations must educate, recruit, and retain health care professionals of diverse backgrounds. Further, these professionals be representative of the diverse Canadian population. Additionally, Phillips and Malone [6] argue that minority nurses have an important role in the healthcare system since they contribute to the recruitment and retention efforts of a diverse workforce. Finally, to truly diversify the nursing workforce, address intra-professional tensions and improve health outcomes for populations, it is essential to both eliminate barriers to accessibility that reinforce exclusion and marginalization, in addition to enhancing the sense of belonging for groups who have historically been marginalized and excluded from nursing [8, 26, 28].

Growing attention to anti-Black racism, particularly as it relates to nursing, reinforces both the timeliness and necessity of this review. There is a need to understand the literature by charting the existing evidence related to Black nurses in Canada. A scan of the literature determined that no other scoping or systematic review on this topic exists. Thus, the purpose of this scoping review was to chart the existing evidence regarding Black nurses in the nursing profession in Canada. This review, which is a component of a larger doctoral research project, offers recommendations for future research regarding Black nurses in Canada. Finally, this review contributes to the international call-to-action by the United Nations, to improve the human rights, social wellbeing, and overall health of people of African descent in Canada and globally.

### Review question and objectives

What evidence exists regarding Black nurses in the nursing profession in Canada? Specifically, to:

1) Describe how Black nurses have been represented in the literature.

2) Map existing evidence to inform knowledge gaps and priorities for future research.

### Inclusion criteria

#### Participants

This review sought studies involving Black nurses in Canada. Black nurses are defined as nurses who reside in Canada and identify as being Black or of African descent. Specifically, Black nurses may include immigrants from continental Africa, the Caribbean or United States; people of African descent who reside in Canada; as well as Black people with ancestral connections to Canada such as African Nova Scotians. No restrictions were placed on other key demographic details such as gender, sexual orientation, class or disability. Studies that did not include participants who were identified as Black nurses were excluded. Furthermore, studies that did not include race-disaggregated findings or results were excluded. For example, studies that focused on internationally educated nurses, which aggregated internationally educated nurses from multiple countries without a clear indication as to which data applied to Black nurses, were excluded.

#### Concept

The concept of interest was the nursing profession, specifically Black nurses in nursing. Thus, sources were considered for inclusion if they referenced an aspect of the nursing profession, including clinical care, education, administration, policy or research. Studies were excluded if they focused on non-nursing care providers [i.e.: physicians, psychiatrists, physiotherapists], nursing students [i.e.: diploma or baccalaureate], non-licensed clinical care providers [i.e.: personal support workers or continuing care assistants], or if the sources aggregated data on various health care providers without clear indication as to which data were nursing specific.

#### Context

The context for the scoping review is Canada. Sources were considered for inclusion if they related to any of the 13 provincial or territorial regions or if the sources were national in scope. Sources that involved multiple countries were considered for inclusion if they included disaggregated data regarding Canada and Black nurses.

#### Types of sources

This scoping review considered all qualitative, quantitative, and mixed methods study designs as well as systematic reviews for inclusion. Gray literature such as dissertations, text and opinion papers, as well as organizational reports or policy documents were considered for inclusion in this scoping review. Finally, no restrictions were placed on date of publication, however, language restrictions were limited to English and French.

## Methods

JBI is an international evidence-based healthcare research organization that is a global leader in the production and dissemination of evidence syntheses. JBI has over 70 collaborating entities globally to promote, support and implement evidence into healthcare practice [29]. Currently, JBI offers formal methodological training and guidance on 10 types of reviews, with scoping reviews being a common approach. Scoping reviews are an effective approach to map or chart a particular area of research, particularly if that area is unclear or poorly defined. These reviews use the mnemonic, *PCC*, which represents *participants, concept,* and *context*. Scoping reviews are an excellent starting point in research as they help to identify types of available evidence in a given field, they facilitate the identification and summation of knowledge gaps, they allow for the clarification of key concepts and definitions in literature, they provide an understanding of how research is conducted in relation to a specific topic or field, and they enable the identification of key characteristics or factors related to a concept. Finally, by clarifying working definitions or conceptual boundaries related to topics or fields, scoping reviews serve as excellent precursors to qualitative, quantitative or mixed methods systematic reviews. To this end, a scoping review conducted in accordance with the JBI scoping review methodology, was the appropriate method of inquiry [29]. The objective and methods guiding this review were published in an a priori protocol [7].

### Search strategy

The search strategy was developed in collaboration with a librarian and peer reviewed by a second librarian using the Peer Review of Electronic Search Strategies (PRESS) [30]. The search, which was conducted on August 31^st^, 2020, aimed to locate published studies and gray literature. An initial limited search of CINAHL was undertaken to identify articles on the topic. Keywords in the titles and abstracts of relevant articles, and the index terms used to describe the articles were used to develop the final search strategy used to search databases from inception to present. The search strategy, including all identified keywords and index terms, was adapted for each included information source. Search terms related to the population of interest included African Canadian, Black, African Nova Scotia, immigrant, and minority, as these terms are used in Canada. Other keywords included nurse and Canada. The search strategy is located in Appendix 1. Ancestry searching and forward citation tracing was performed to identify relevant sources. Lastly, searches were restricted to literature published in Canada’s two official languages, English and French.

### Information sources

Information database sources include CINAHL (EBSCO), MEDLINE (Ovid), Embase (Elsevier), Sociological Abstracts (ProQuest), Gender Studies Database (EBSCO), America: History and Life (EBSCO), PsycINFO (EBSCO), Academic Search Premier (EBSCO), and Scopus (Elsevier). Sources of unpublished studies and gray literature searched included websites of the Canadian Nurses Association, Registered Nurses Association of Ontario, College and Association of Registered Nurses of Alberta, Nova Scotia College of Nursing, and ProQuest Dissertations and Theses Global (ProQuest). Scopus (Elsevier) was also used for the forward citation tracing.

### Study selection

Following the search, all identified citations were collated and uploaded into Covidence (Veritas Health Innovation, Melbourne, Australia) and duplicates removed. From the title/abstract stage through to data extraction phase, each study was reviewed by two independent reviewers. At the title and abstract, full-text, and data extraction phases, several articles were screened by two independent reviewers against the inclusion criteria in a pilot test to calibrate the screening and extraction tools [7].

**Fig. 1:**
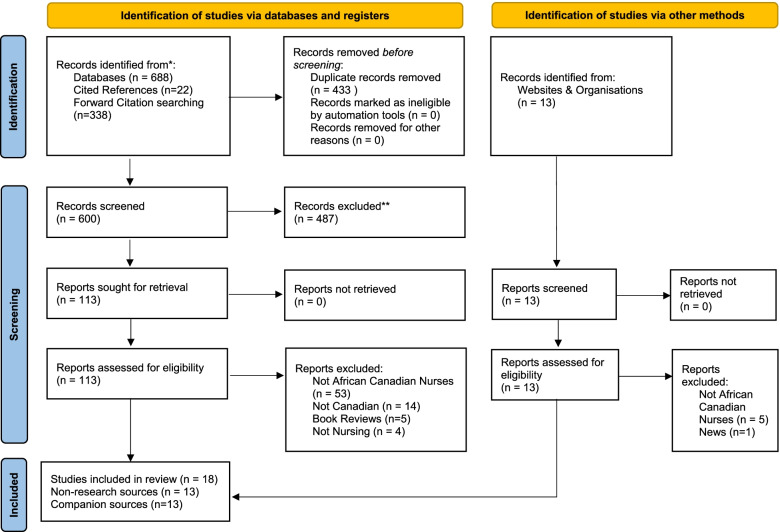
PRIMSA-ScR Flowchart of Study Selection Process. *Consider, if feasible to do so, reporting the number of records identified from each database or register searched (rather than the total number across all databases/registers). **If automation tools were used, indicate how many records were excluded by a human and how many were excluded by automation tools. *From:* Page MJ, McKenzie JE, Bossuyt PM, Boutron I, Hoffmann TC, Mulrow CD, et al. The PRISMA 2020 statement: an updated guideline for reporting systematic reviews. BMJ 2021;372:n71. https://www.doi.org/10.1136/bmj.n71. For more information, visit: http://www.prisma-statement.org/

At the title and abstract and full-text screening stage, items that did not meet the inclusion criteria were excluded. Specifically, sources were screened first according to the context, the next level of screening was based on participants and the final level of screening was the concept. Reasons for exclusion of full text studies not meeting the inclusion criteria were recorded and are reported in Appendix 2. Lastly, in accordance with the a priori protocol [6], any disagreements that arose between the reviewers at each stage of the study selection process were resolved through a third reviewer or through discussion with the review team. The results of the search are reported in Fig. 1, in the Preferred Reporting Items for Scoping Reviews (PRISMA-ScR) flow diagram [31].

### Data extraction

The data extraction tool, appended in the a priori protocol [7], was developed based on a JBI standard extraction form. This extraction form was imported into the Covidence software and extraction was completed independently by the first author and a second extractor who was a member of the review team. Conflicts or disputes that arose between the reviewers, regarding inclusion/exclusion of sources or data extraction, were resolved by a third reviewer or through discussion with the review team.

### Data items extracted

Specific data items for which data were sought are found in the extraction tool appended in the a priori protocol [7]. Examples of data items included: source title, year of publication, and source type. Additional data items related to methodology included the: aim/purpose, questions/objectives, study design, and sample size; and additional data items included results/key findings with accompanying quotations and statistics relevant to the review objective. Finally, the set of associated assumptions regarding the participants, concept and context were as follows:

### Participants

It is assumed that all Black nurses in Canada have completed nursing training at either the diploma or baccalaureate level. It was also assumed that the use of the term *Black* referred to people of African descent, who identified as such. Conversely, this review did not assume that studies that described participants as visible minority, immigrant, racialized, etc. were exclusively Black nurses. Yet, this review did assume that nurses identified as immigrating from the Caribbean and continental Africa were Black. This assumption was not made without careful consideration of the population, sample and demographic details provided by source authors. Finally, all participants were assumed to be adults, over the age of 18.

### Concept

Assumptions about nursing included the restrictions regarding licensing and registration in nursing as well as the criteria related to the use of the title *nurse*. For example, sources that referred to nursing, unless otherwise stated, were assumed to involve practical nurses, registered nurses and/ or advanced practice nurses. In Canada, the term *nurse* is a protected term that cannot be used by non-nursing care providers. The concept of nursing extended to include any practice setting. While any sources that involved pre-licensure nursing students were excluded.

### Context

In terms of context, an assumption around language was made. The search was restricted to French and English, which are the two official languages in Canada.

### Analysis and presentation of results

Data from included sources were extracted and stored in Covidence. After extracting data in Covidence, data were then exported and managed in Microsoft Word. Data were reviewed and organized, by team members, using Microsoft Word in addition to hand-written notes. Microsoft Word and hand-written notes were used to generate the categories into which sources were sorted. The approach for summarizing and presenting findings, through the creation of categories is described in the a priori protocol [7]. The presentation of results is done in an appropriate manner to facilitate mapping the existing evidence regarding Black nurses in Canada. Thus, the data are presented narratively in categories as well as in diagrammatic or tabular form, where appropriate. The presentation of the review results aligns with the scoping review question and objectives for charting the existing evidence regarding Black nurses in the nursing profession in Canada. Additionally, the review categories that depict the findings were not developed through a thematic or qualitative process. Rather, the categories are derived directly from the included sources and serve only as a means to classify each source. Finally, a critical appraisal of sources was not conducted as this review is scoping in nature and should not be conflated with or regarded as a qualitative systematic review.

## Results

### Study inclusion

The search of databases yielded 688 records with 338 identified through citation tracing. After removing duplicates, 600 titles and abstracts were screened for eligibility and 127 advanced to full-text screening. From this process, 82 full-text articles were excluded based on inclusion criteria. Appendix 2 includes a list of excluded sources and accompanying rationale for exclusion. A total of thirty-seven (*n* = 37) [32–68] sources were retrieved through the database search and seven (*n* = 7) [69–75] sources were identified through the gray literature search. Subsequently, 44 [32–75] sources met the inclusion criteria and 31 [32, 35–42, 50, 53–64, 66, 68–75] of these sources underwent data extraction. The PRISMA-ScR flowchart in Fig. 1 shows the study selection process.

**Fig. 2:**
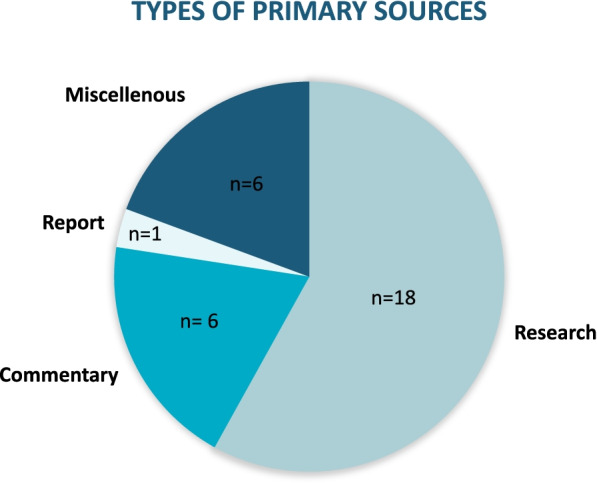
Type of Primary Sources Included

The 44 sources that met the inclusion criteria are classified into two distinct groups: primary sources (*n* = 31) [32, 35–42, 50, 53–64, 66, 68–75] and companion sources (*n*= 13) [33, 34, 43–49, 51, 52, 54, 65, 67]. Primary sources are defined as original, stand-alone sources, which do not share data with other sources. Companion sources include sources (commentaries and research reports) that were retrieved through the systematic search and met the inclusion criteria however, they use data from one of the included primary sources. It was necessary to group the included sources in this manner to avoid the duplicate extraction of data. Figure 2 depicts the type, and frequency, of primary sources included in the review.

In this review, only the primary sources (*n* = 31) [35–42, 50, 53–64, 66, 68–75], underwent data extraction. This decision was made to avoid confusion by the inclusion of duplicate data from companion sources (*n* = 13) [33, 34, 43–49, 51, 52, 54, 65, 67] using data from a primary source. A description of the 31 included sources that underwent data extraction is located in Appendix 4. The companion sources, which are detailed in Appendix 5, did not undergo data extraction and thus are not included in the results section. The process for selecting the primary source, in instances when multiple sources were retrieved, was based on two criteria: 1) a comprehensive and thorough description of study design and methods, and 2) consistency amongst sources. For example, if there were multiple sources that were generated from an included dissertation, the dissertation was selected as the primary source. There is one exception, where the dissertation is classified as a companion source and the book generated from this research was selected as the primary source [50]. The four primary sources from which the companion sources arose are outlined in Table 1.

**Table 1:** Primary sources and companion sources

Primary sources with companion sources—Research Studies (*n* = 4)	Companion sources—Commentaries and Research (*n* = 13)
Boateng (2015) [32]	Boateng (2016) [33] Boateng (2019) [34]
Etowa (2005) [42]	Etowa (2006) [43] Etowa (2007) [44] Etowa (2009) [45]
Flynn (2011) [50]	Flynn (2003) [46] Flynn (2004) [47] Flynn (2008) [48] Flynn (2009) [49] Flynn (2012) [51] Flynn (2015) [52] Shkimba and Flynn (2004) [65]
Hagey (2001) [55]	Turrittin (2002) [67]

### Characteristics of included sources

Of the 31 primary sources included in this review, the year of publication ranged from 1993 to 2020. All included sources were published in English except one (*n*= 1), which was published in French [35]. Another source focused on Francophone-African nurses [59]. All participants were adults, over the age of 18. In terms of gender, fourteen sources focused on participants identified as women (*n*= 14) [35–38, 40, 41, 46, 50–55, 57, 60, 62, 66], while five (*n* = 5) [32, 42, 59, 61, 63] included both women and men and twelve (*n*= 12) did not specify gender [39, 56, 58, 64, 68–75].

Eighteen of the 31 primary sources are classified as research studies (*n* = 18) [32, 35–38, 40, 42, 50, 55, 57, 58, 60–64, 66], of which five (*n* = 5) [32, 42, 46, 62, 66] are doctoral dissertations and two (*n* = 2) [41, 50] are books. Fourteen of the eighteen research studies were qualitative designs (*n*= 14) [32, 35–38, 40, 42, 50, 55, 57, 60, 62, 63, 66], in which data collection methods included interviews (*n* = 10) [32, 37, 38, 50, 55, 57, 60, 62, 63, 66] or a combination of methods such as document/ literature review, interviews, group discussions and observation (*n*= 4) [35, 36, 40, 42]. One (*n* = 1) [61] of the eighteen studies employed a quantitative study design, as a secondary data analysis and two (*n* = 2) [41, 58] studies used a mixed methods design, which used a combination of interviews and secondary analysis. Finally, one (*n*= 1) study was a systematic scoping review [64]. The theoretical frameworks or methodologies for the research studies included critical social approaches, such as integrative anti-racist frameworks, Black feminist theories, postcolonial feminist perspectives, and intersectionality [37, 50, 55, 62, 63, 66]. Others employed more traditional methodologies including phenomenology, critical ethnography, grounded theory or descriptive [35, 38, 42, 57].

### Sources by geographical area

The context for this review was Canada thus, all 31 included sources were from within Canada. Sources display regional trends, with the majority of sources being concentrated in the province of Ontario (*n*= 14) [32, 35, 38–41, 55, 60, 62, 68, 71–75]. Additional locations included Nova Scotia (*n*= 2) [42, 57], Manitoba (*n* = 1) [59] and Quebec (*n*= 2) [53, 63]. While 10 (*n* = 10) [36, 37, 50, 56, 58, 61, 64, 68–70] sources included multiple provinces or were national in scope.

### Review findings

**Fig. 3:**
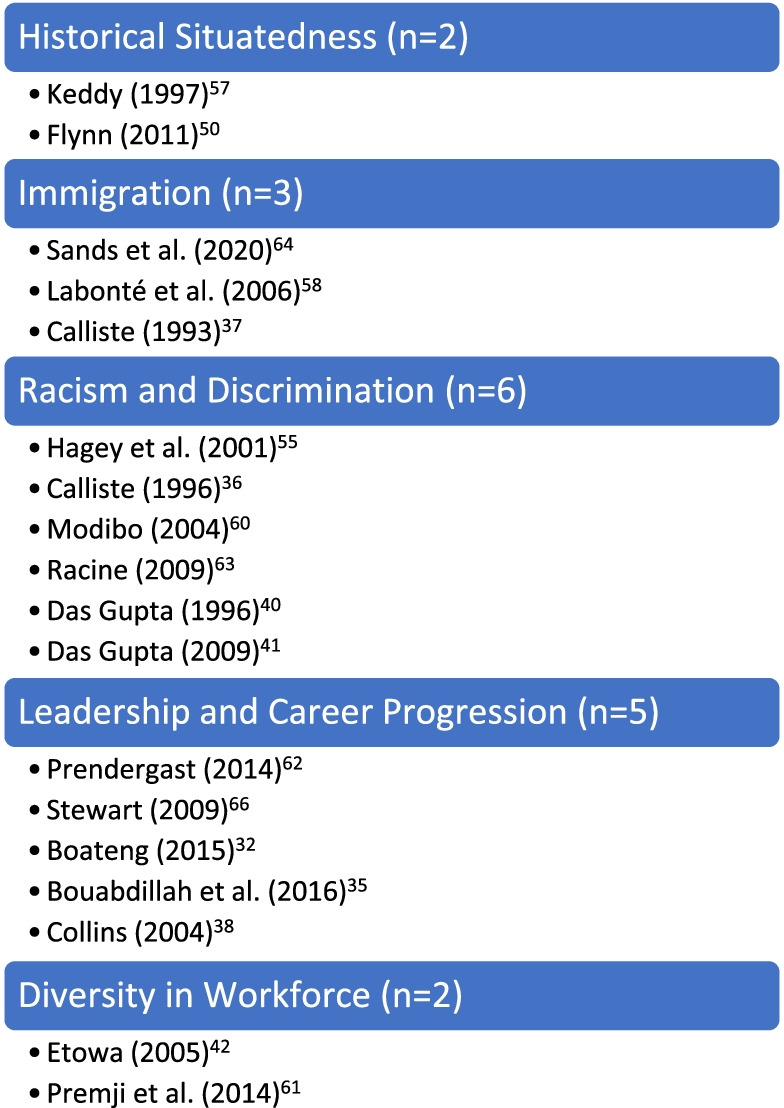
Categories in Nursing – Research Studies (*n* = 18)

**Fig. 4:**
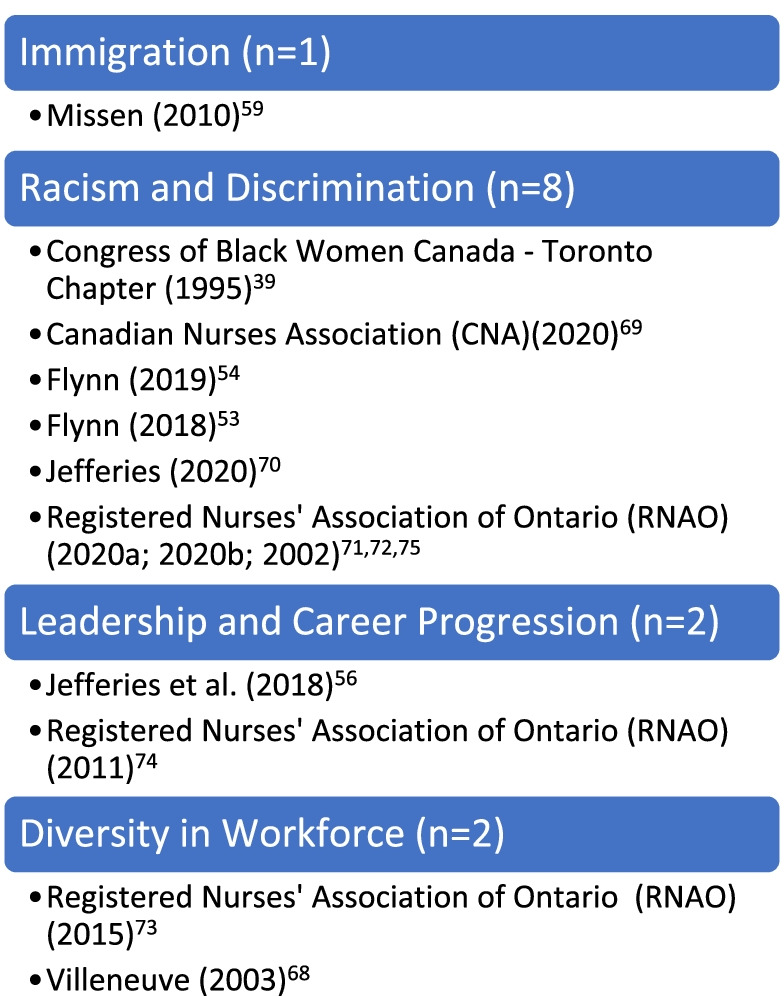
Categories in Nursing – Non-research Sources (*n* = 13)

As noted in the a priori protocol [7], the purpose of this scoping review was to chart existing literature regarding Black nurses in the nursing profession in Canada. The results of this review are presented according to five conceptual categories generated from the 31 primary sources. These categories were generated by identifying the aim or purpose of the source, which generally included a central concept such as *racism in nursing*, *leadership* or *immigration*. It is important to note that the nature of this review inevitably resulted in an overlap of key findings across categories. Ergo, sources were classified under the category based on terminology used in the title of the source as well as in the aim or purpose. It is acknowledged that an argument could be made for a number of sources to be classified differently. With this in mind, the presentation of the results of the scoping review should be viewed as intended – a mapping or charting of existing evidence. Figures 3 and 4 illustrate the classification, and the number of sources within each, for the research and non-research sources, respectively. The sources in each of these categories are further detailed in Appendix 3. As shown below, the categories include: historical situatedness; immigration; racism and discrimination; leadership and career progression; and finally, diversity in the workforce. To reiterate, this scoping review maps the existing evidence regarding Black nurses in the nursing profession in Canada. It is not a qualitative review thus, there is no interpretive component nor critical appraisal of methodological quality for the included sources.

### Historical situatedness

This category focused on situating the historical experiences of Black nurses in Canada. While multiple sources described salient moments pertaining to the historical context, two [50, 57] research studies explicitly focused on describing the historical experience of Black nurses in Canada. These two sources used a combination of oral histories and archival sources to document the experiences of Black nurses in Canada. Reviewing and documenting the historical accounts of Black nurses in Canada revealed repeated attempts to erase the contributions and presence of Black nurses [50]. However, despite the historical exclusion and attempted erasure of Black nurses from various records, researchers succeeded in uncovering these records, documenting stories, identifying issues and addressing conspicuous gaps in nursing history [42, 50, 56, 57, 70]. For example, Flynn’s [50] review of archival documents and records revealed that prospective Black students were not permitted to enter Canadian nursing training institutions until the 1940s. Flynn found that the refusal to admit Black students into nursing was done on the basis of racial discrimination and prejudice, with training facilities citing race-related factors as the reason for denial of admissions.

The use of oral histories from Black nurses illustrated a portrait of leadership that shed light on social issues that shaped contemporary nursing. Keddy [57] found that Black nurses in the province of Nova Scotia had professional experiences that differed significantly from those of white nurses. Moreover, the historical account, generated by Flynn [50] and Keddy [57], explains the similarities and difference amongst Canadian-born Black nurses and Black IENs. Both Keddy [57] and Flynn [50] shared how Canadian-born Black nurses described the impact of socialization in a predominantly white society, where experiences of racism and segregation began in childhood. Conversely, Black nurses who immigrated to Canada from Africa or the Caribbean, as IENs, reported challenges with national immigration policies in addition to cumbrous nursing registration and licensing requirements that delayed integrating and acceptance into the profession and the country.

### Immigration

In the context of this review, immigration refers to the formal processes of people of African descent migrating to Canada. Three research studies [36, 60, 64] and one commentary [59] described the immigration or migration patterns of Black nurses to Canada. The Caribbean and continental Africa were noted as being amongst the most prevalent countries of origin for Black IENs [36, 60, 64]. Complex systemic processes and policies were shown to have a significant impact on the migration patterns of IENs. Restrictive immigration policies paired with poorly defined or unclear processes were found to be recurring obstacles for IENs. Immigrating to Canada, for Black IENs, was aggravated by significant challenges with integration into the nursing profession, consequential to navigating the licensing and registration process. For example, Francophone-African nurses immigrating to Canada spoke about navigating a complex and unclear immigration process, adapting to more “individualistic cultural” norms as well as enhanced feelings of safety and security in Canada [59]. For the IENs who successfully integrated into Canada and the nursing profession, there were accounts of additional issues and challenges. Boateng [38] described the systemic issues that were encountered by IENs and visible minority nurses in their career pathways and immigration into Canada. It was found that IENs encountered more systemic issues within the workplace, more verbal abuse, were excluded from upward career mobility and were denied the opportunity to be a part of decision-making processes [38]. Additionally, IENs had a more indirect, convoluted, and lengthy pathway into the nursing profession [32].

A review of literature, interviews and the analysis of official documents and policies showed that Canadian immigration policies effectively restricted and limited the entry of professional and skilled workers from the Caribbean [36, 64]. One such policy, entitled *Women of Exceptional Merit*, required that Caribbean nurses demonstrate a level of merit, which far exceeded the professional qualifications of non-Black nurses, to gain entry to Canada [36, 64]. This policy dictated that Black nurses from the Caribbean could be granted temporary entry to Canada if they were deemed to be of “exceptional merit” and with a promise to return to their home countries after their transitory stay [36, 64]. *The Women of Exceptional Merit*policy created systemic barriers in regulatory practices and work environments, which Caribbean nurses were forced to navigate [64]. Each of the studies that examined immigration and migration argued that there is an urgent need to evaluate and revise immigration policies, especially as the migration of Black nurses into Canada accelerates [36, 60, 64].

### Racism and discrimination

Racism and discrimination are terms used to describe the intentional or unintentional stereotypical assumptions and negative treatment based on various factors such as race, gender, and class. Racism and discrimination were frequently occurring central issues identified in six research studies [37, 40, 41, 55, 60, 63] and eight non-research sources [39, 53, 54, 69–72, 75] related to Black nurses in Canada. Four of the six sources included discriminatory lawsuits or grievances filed by Black nurses in the provinces of Ontario [40, 41, 55] and Quebec [53]. Racism and discrimination were described as multifaceted issues that occurred interpersonally as well as systemically [37, 40, 41, 55, 60, 63]. These deeply embedded issues were found to permeate multiple levels, from everyday interpersonal interactions to institutional processes and policies as well as attitudes and ideologies. In terms of interpersonal experiences of racism, Black nurses encountered racism perpetrated by patients, colleagues and supervisors [39, 40, 55, 60, 63]. Black nurses reported being subjected to racial slurs in addition to microaggressions relating to their appearance, heritage or ability to perform their work [60]. For example, Black nurses described everyday workplace experiences that included issues of differential treatment noting that they did not receive standard professional courtesies [60]. Black nurses also reported mistreatment and verbalized racial abuse from patients, including some patients refusing to be cared for by “Black hands” [60].^(p.111^^)^

Systemic racism included discriminatory institutional and organizational policies, procedures, and processes. Hiring processes, unjustified termination, enhanced surveillance, and lack of organizational support were reported as elements of systemic racism [37, 40, 41, 53, 55, 60, 63]. Calliste [37] explained how systemic issues including economic restructuring (in the form of lay-offs) disproportionately impacted Black nurses. Additionally, Black nurses highlighted systemic racism in the form of discriminatory hiring practices [53, 63]. Sources that examined the legality of formalized grievances and lawsuits determined that racism was a precipitating factor in all of these proceedings. In one instance, complaints filed with the Ontario Human Rights Commission (OHRC) found that two Black nurses had experienced racism that was gendered and classed [40]. This finding introduce the notion of intersectionality, which occurs when discrimination is attached to multiple social constructs such as being a woman, racialized and an immigrant [37, 40, 63]. Several sources in this review described the racially specific gendered and classist ideologies that were used to justify the racial division of labor, exploitation, and the devaluation of Black nurses [37, 40, 63].

Nurses who filed grievances due to discrimination by their employers also reported feelings of marginalization, experiencing physical stress and emotional pain, in addition to the need to develop strategies to cope [55]. Moreover, there was a high degree of fear, lack of support, harassment and ineffective institutional responses, which made it difficult for Black nurses to report and take action against racism and discriminatory practices [41, 55]. A landmark case involving Gloria Baylis provides another example of a racial discrimination case in nursing in Canada related to discriminatory hiring practices. The Baylis case [53], like others, attracted media attention and contributed to the shift towards addressing issues of racial discrimination in nursing and society.

Finally, the ongoing issue of racism and discrimination in the nursing profession led two prominent nursing organizations in Canada to draft and share statements denouncing anti-Black racism in nursing in Canada [69, 71, 72, 75]. The Canadian Nurses Association (CNA) identified anti-Black racism as a public health crisis, acknowledged the legacy of anti-Black racism in Canadian nursing history, and described ways that CNA intended to combat anti-Black racism [69]. While the RNAO, which has challenged discrimination for decades, released a policy statement detailing their stance against racism in nursing [75]. The policy outlined their commitment to creating an environment where all nurses and patients are valued and treated with respect and dignity. These public organizational statements, which denounced anti-Black racism, are important messages considering the pervasiveness of racism especially since racism was identified and described as a significant determinant in career progression, advancement, promotion and entering formal leadership roles [32, 35, 39, 56, 62, 66, 74].

### Leadership and career progression

Leadership and career progression, within the context of this review, refers to the vertical and lateral movement within the nursing profession. Career pathways, promotions, professional development, upward movement, opportunities for advancement and obtaining formal leadership roles, were examples of vertical and lateral mobility used to describe career progression and leadership [32, 35, 38, 62, 66]. Five research studies [32, 35, 39, 62, 66] and two non-research sources [56, 74] were classified in this category. The definition of leadership varied across included sources, with some sources referring to leadership exclusively as formal roles or positions such as managers [35, 66]. Sources highlighted systemic barriers to career advancement and mobility, underrepresentation in leadership and managerial roles, job dissatisfaction and a lack of opportunities to support and facilitate advancement. Again, as aforementioned, racism was identified as a significant determinant in career progression, advancement, promotion, and entering formal leadership roles for Black nurses [32, 35, 39, 56, 62, 66, 74].

The notion of career progression, promotion and mobility was interwoven with many sources that discussed leadership. Despite studies examining leadership, Black nurses were found to be underrepresented in management roles [35, 61, 70]. The impact of the exclusion of Black nurses from leadership positions was intensified by Black nurses having heavier workloads [35, 39]. Collins’ [38] determined that IENs experienced more systemic issues within the workplace, were excluded from lateral and vertical career mobility opportunities, were excluded from decision-making processes, and had repeated negative workplace interactions including verbal abuse. Effectively, the career trajectory of Black nurses was fraught with discrimination [35]. For example, the underrepresentation of Black nurses in leadership positions was described in unison with feelings of exclusion from attaining management positions [35]. The nurses expanded upon their interpretation of systemic barriers to explain how collegial relationships and workload also impacted their career advancement. A lack of managerial support, heavier workloads, and collegial conflict were perceived as barriers to promotion. Notably, white nurse managers acknowledged the underrepresentation of Black nurses in leadership roles but felt that the process of career promotion and advancement in nursing was both fair and transparent [35].

When present, Black nurse managers reported feeling undervalued, marginalized, isolated, receiving negative differential treatment, experiencing criticism and needing to work twice as hard as white colleagues [56, 66]. Specifically, Stewart’s [66] investigation of the impact of race on the work experiences of Black women nurses in formal leadership positions found that these nurses experienced unfair treatment based on race; a lack of guidance and support from peers; feeling invisible or unimportant; the need to prove oneself as a leader by being ‘twice as good’; and the need to negotiate racial identity as a benefit or liability [66]. In addition, Black nurse managers expressed decreased job satisfaction due to these experiences of everyday racism, microaggressions and negative criticism [66]. An examination of multiculturalism policies in nursing revealed that Black IENs tend to occupy a hybrid space, involving minimal leadership responsibility with no movement into higher levels of leadership [58]. IENs entered this hybrid space due to their multicultural and multilingual abilities being viewed as assets. Yet, despite these assets, IENs expressed that their work went unrecognized and that they were excluded from policy-making leadership positions. This realization was compounded by the absence of Black nurse leaders in formal leadership positions [62]. Remarkably, Prendergast [62] identified an “ideal type” in nursing leadership related to those who occupy policy-making positions, who were found to be white, middle-class nurses.

### Diversity in the workforce

As described in the a priori protocol, diversity in the workforce refers to the active effort to have a profession that includes individuals from a multitude of groups who bring experiential knowledge, insight, and variable difference across aspects such as gender, race, age, sexual orientation, language and ability. The importance of promoting and cultivating diversity in the nursing workforce was a resounding theme present in multiple sources throughout this review. In terms of diversity in the workforce, there are two research studies [42, 61] and two non-research sources [68, 73] that were classified under this category. Etowa’s [42] grounded theory describes how Black nurses navigated and succeeded in nursing while feeling as though they were practicing on the margins of the profession. Nurses felt that despite being an insider by virtue of their education, training, and values, there was an ever-present struggle to navigate a profession in which they considered themselves as practicing outside the center. Key internal and external drivers were identified as necessary to facilitate integration into the profession [42]. Combined with the diminished sense of belonging in the profession, a diversity profile of Canada’s nursing workforce, using census data, found that visible minorities were concentrated or over-representated in entry-level nursing positions [61]. Additionally, linguistic minorities were found to be underrepresented in all areas of the nursing profession. The linguistic component of the nursing workforce is of particular interest in nursing since there is a sizable number of Black nurses who speak French (or another language) as their first language, including those from Francophone-African countries who have reported additional discrimination based on language or accent.

In recognizing the underrepresentation of people of colour in nursing leadership, Villeneuve highlighted the importance of diversifying Canada’s nursing workforce through race, gender, and age [68]. Achieving a truly diverse nursing workforce was described as requiring multilevel action from local, provincial and federal government and healthcare organizations including an acknowledgment of issues; a commitment to diversity; conducting race-based research; modifications to nursing curricula and admission policies; the removal of arbitrary barriers hindering IENs from practicing in Canada; and nurturing leadership and career progression [68]. Premji and Etowa [61] reverberate this sentiment in that there is a need for increased diversification in nursing, which can be facilitated by the inclusion of linguistic and visible minority nurses in higher level leadership positions. However, culturally- and linguistically focused initiatives alone are cautioned against, as these initiatives without proper scaffolding, are insufficient in a sustainable reduction of health disparities. Rather, health system reform, including social and economic policies to complement directives, is required to promote diversity in nursing and benefit multiple sectors [61].

### Summary of review findings

**Fig. 5:**
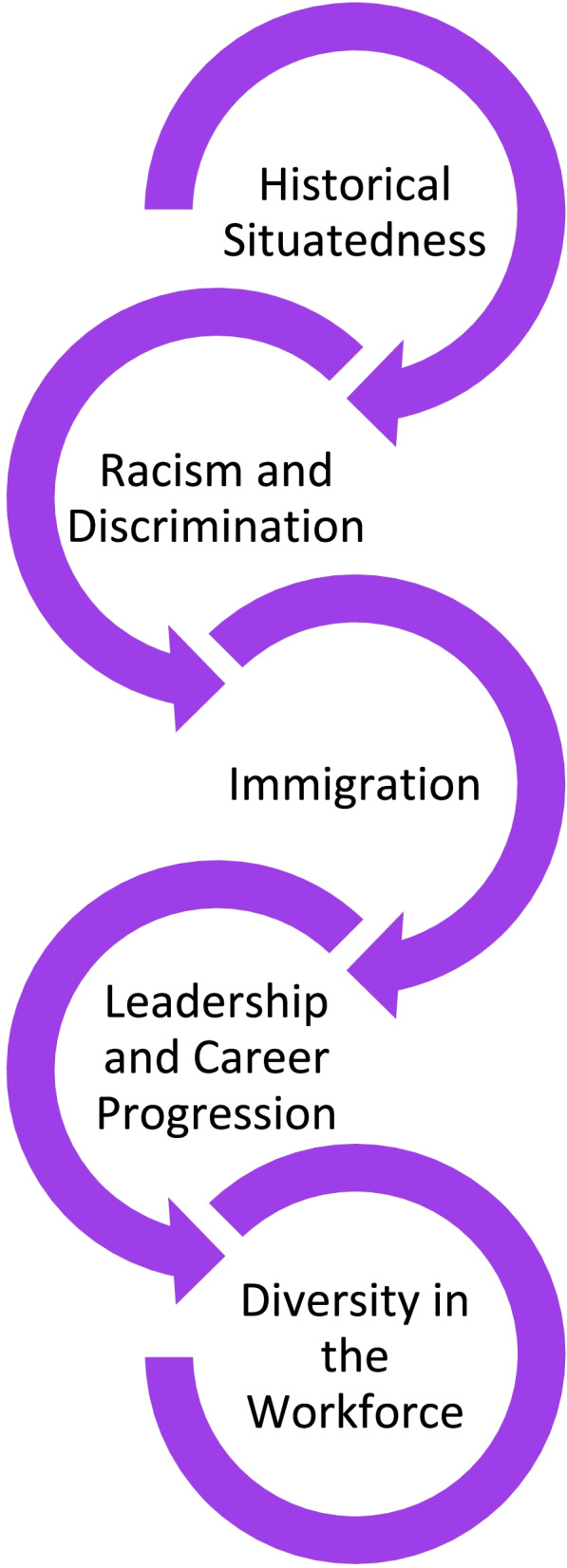
Relationship Between the Five Categories

The results of this review reveal an interconnected relationship between the five main categories identified in the literature. This review shows how critical concepts, such as immigration and/or diversity in the workforce, can be traced to the historical situatedness of Black nurses in Canada. Specifically, racism and discrimination, which are manifested through institutional policies, systems, procedures, and interpersonal interactions, reinforced and perpetuated the underrepresentation of Black nurses in the workforce and in leadership positions. Further, despite the results being presented in five overarching categories, many of the included sources overlapped significantly with regards to the five identified categories. For example, the most prevalent category (racism) was woven throughout the majority of the sources in this review. The dominance of racism as a central theme across the majority of sources raises important questions regarding the nature of the literature, the experiences of Black nurses in Canada, and the nursing profession overall. Figure 5 offers a visual to demonstrate how each of the five categories are interconnected and linked to one another.

## Discussion

The findings from this review provide a comprehensive overview of the current available evidence that exists pertaining to Black nurses in Canada. The results are presented in relation to the review question and objectives described above and in the a priori protocol [7]. The specific categories generated from this review include historical situatedness; the immigration; racism and discrimination; leadership and career progression and finally, diversity in the workforce. The following discussion provides a succinct recap of the review findings by situating the review findings amongst a larger body of literature and offering an interpretation of the findings. After which, implications for future research are presented by considering critical aspects including how knowledge of history provides insight into the current circumstance in addition to direction for the advancement of nursing in Canada through the mobilization of Black nurses.

### Using the past to understand the present and to inform the future

This review charts the evidence by situating the historical context for Black nurses in Canada. Uncovering the historical record of integration for Black nurses into the nursing profession in Canada, from a critical and intersectional perspective, reveals the ways in which anti-Black racism in nursing excluded Black people from the profession [9, 50, 70]. Flynn described how Victorian ideals of “true womanhood”, including ideals of femininity, purity and respectability were operationalized to not only discriminate against individuals and groups based on gender, race, sexual orientation, ability and class but to also dictated *who*could become a nurse [9, 50, 70]. These restrictive ideological standards restricted entry into nursing, and were juxtaposed in direct opposition to stereotypes attached to Black women [50, 70, 76]. The continued repeal of these restrictive discriminatory admissions standards in nursing is largely credited to community activists and organizations that were vocal and active in challenging racism and discrimination in Canadian nursing training programs. For example, as described, Oliver and the NCAAP are credited as being a catalyst by playing a pivotal role in challenging discriminatory admission practices in nursing in order to facilitate the entry of Black women into nursing programs in Canada [9, 15].

### Situating within the literature

Much of the research included in this review classifies Black nurses in Canada into two groups: Canadian-born Black nurses and Black IENs. While there is a larger breadth of literature associated with Black IENs in Canada compared to Canadian-born Black nurses, the phenomena of Black nurses in Canada remains understudied. Furthermore, Sands et al. [64], as well as other scholars, caution against the temptation to aggregate the experience of “all Black nurses” as this blunder tends to mask the nuances that exist within the Black population and warrant more in-depth consideration. To this end, much of this discussion situates the review findings around Black IENs. However, this highlights another significant gap in the existing literature, which will be address in the implications for research section.

This review highlighted the various policy-related challenges that Black IENs encountered both with immigration to Canada as well as with integration into the nursing profession [77, 78]. Professional licensing and registration have reportedly taken years for IENs to have their credentials recognized. During this time, many IENs practice in non-licensed clinical roles, such as continuing care assistants or personal support workers. While others, who are educated and trained as specialists, registered, or advanced practice nurses, occupy entry-level or practical nurse roles [79]. Additionally, this review describes the interprofessional challenges that Black IENs in Canada encountered when attempting to integrate into the nursing profession. Collegial issues, tension with management, being treated as an outsider and negative encounters with patients, including physical and verbal abuse, were common issues [80]. Whereas, good collegial relationships as well as opportunities to enhance nursing knowledge and skills were shown to have a statistically significant positive association with workplace integration [77]. Everyday work experiences and integration into nursing, corroborate the work of O’Brien Pallas et al. [81] who found that the IEN workforce tended to work longer hours (including overtime) and were more likely to experience physical and mental abuse. O’ Brien Pallas et al. [81] explain that self-rated physical and mental health was lower for IENs, especially as they experienced inequity in learning opportunities as well as job instability. However, despite these issues, IENs perceived themselves as an asset to the nursing profession, especially in terms of linguistic diversity and cultural practices, which was similarly found in this review [82].

Literature shows that IENs entering nursing in Canada are faced with a distinct set of obstacles compared to Canadian-born Black nurses [50, 75, 83]. Uniquely challenging to IENs is the task of bridging international certification to practice nursing in Canada. According to Blythe and Baumann [83], IENs have a later start to their nursing careers in Canada due to issues navigating educational upgrading and language requirements. Similar findings were uncovered in this review as restrictive immigration policies posed significant barriers for Black IENs who sought to migrate to Canada [32]. Yet, similar to Canadian-born Black nurses, IENs experienced racism and discrimination, which reduced opportunity to integrate into the profession and enter leadership positions [83]. This captures the manner in which multiple factors influence and impact experience. Intersectionality is an analytical framework that interrogates and challenges systems of oppression that have historically led to the exclusion of factions of society based on nationality, disability, race, class, gender, sexual orientation, age and other social constructs [84, 85]. This review captures how the process of immigration and integration is structured and controlled by social constructs including race, class and gender [36]. Again, intersectional experiences of discrimination related to race, class or gender, created significant variations amongst IENs across age, gender, work status, type of work and area of practice [77, 83]. For example, statistical data [77] showed that IENs in Ontario were largely found to settle in more urban centres, which is corroborated by findings in this review. Among included sources, Ontario, which has the largest population of Black people in Canada, contained the majority of the sources regarding Black nurses in Canada. Further, the results of this review show the ways in which the historical foundation of racism and discrimination in nursing has embedded deep-seated ideologies that continue to govern processes and policies in contemporary nursing. Several included studies discussed intersectionality and the experience of various forms of discrimination encountered by Black nurses [37, 40, 53]. While, multiple included studies integrated an intersectional analytic framework to understand the experiences of Black nurses in Canada. For example, uncovering how experiences of racism are compounded and further complicated by gendered and class oppression, were connected to the ways in which Black women were devalued and exploited due to gender ideologies of womanhood [33, 37, 40, 53]. These social ideologies that established a foundation of exclusion of Black women in nursing, and the broader society, continue to be perpetuated in nuanced discriminatory ways [50, 70, 84–86].

Attending to anti-Black racism in interpersonal, institutional, and systemic dynamics is an important consideration to address the nursing workforce shortage. Additionally, the integration of Black IENs has profound implications for the worsening nurse workforce shortage. As explained by Tomblin Murphy et al. [21], addressing the workforce shortage through recruitment, retention, and deployment of nurses, is a useful approach. Therefore, examining the current policies and practices for the integration of IENs in the nursing workforce is critical. Moreover, eliminating racism and discrimination will contribute to efforts to address the lack of diversity in the nursing workforce and in leadership. This process of eliminating systemic and institutional racism is suggested to have ramifications extending into pathways that feed into the workforce since the underrepresentation of Black students in nursing programs is considered a major contributor to issues of representation in nursing [50, 57]. Finally, attention to systemic processes is necessary to establish effective and efficient ways for filing grievances and complaints that do not result in fear of reprisal or ostracization.

Another interesting finding in this review pertains to leadership. As was described in the literature, there was a glaring underrepresentation of Black nurses in formalized leadership roles in Canadian nursing. However, the results of this review depict varying levels of informal leadership that Black nurses displayed through their activism and nursing practice. Duncan [87] suggests that it is imperative that nursing leadership in Canada begin from a place where social justice is used as a framework to guide decision-making, policies, and political advocacy, to ensure health equity on a global scale. Increased diversity in clinical nursing can mitigate health disparities, such as health care access for minority populations, by increasing community-oriented delivery models of health care and nurse-managed primary care [6, 88]. Importantly, transitioning into leadership roles requires a level of readiness from both a personal and organizational perspective. Similar to sentiments expressed by Black nurses in many of the included studies, there must be an investment in development opportunities to support nurses as they transition into leadership positions.

### Black nurses social movement and mobilization

In response to continued anti-Black racism in nursing as well as the heinous killings of Black persons in North America, especially during 2020, Black nurses in Canada have become more vocal about the collective experience of Black nurses and the health of Black people in Canada. Dominant social issues afflicting the Black population served to ignite several collectives and budding networks that were in their early stages of development. The mobilization of Black nurses in Canada has led to collectives forming across the country to create change at the regional, provincial and national level. At the provincial level, current established collectives include the Association of Black Nurses (Nova Scotia), the Ontario Black Nurses’ Network (Ontario), and the Coalition of African, Caribbean and Black Nurses in British Columbia (British Columbia). While the Canadian Black Nurses Alliance (CBNA) and the Pan-Canadian Association of Nurses of African Descent are national organizations that strive to unite Black nurses across Canada. These collectives mobilize at a regional level and engage in intra-provincial collaborations to advance nursing.

### Recommendations for research

Through the mapping of existing evidence regarding Black nurses in the nursing profession in Canada, our review reveals several implications for research. In terms of research design, the majority of research studies included in this review were qualitative. This indicates opportunity for future research that uses a variety of designs and methods outside of the qualitative realm to understand this phenomenon through other paradigms, including the use of measures or tools that incorporate an intersectional analysis. Therefore, future research using quantitative or mixed methods would serve to further inform this phenomenon. An intersectional framework was used in several of the included sources, as authors described the ways in which racism was exacerbated by gendered and class oppression. In addition to applying an intersectional framework to understand the dynamics of race, class, nationality, and gender, it would be useful to examine the ways in which disability, sexual orientation and other critical factors impact Black nurses in Canada.

In addition, a number of the sources included were located in Ontario, which is the most populous and diverse province in Canada. As a result, we suggest expanding the geographical reach to include Western Canada, the Atlantic region, the prairies and Northern Canada. A large, national or multi-provincial study that includes a mixed method design is highly recommended, especially since these demographic data pertaining to the Canadian nursing workforce do not include other key indicators. Additionally, there is a substantial body of qualitative literature (*n* = 18) that would benefit from a systematic review of this evidence. There is an opportunity to review the included primary sources and consolidate the myriad policy recommendations that were produced in many of the research studies. Likewise, a systematic review that examines the companion sources identified in this scoping review would provide greater insight. Additional research of interest may include text and opinion reviews as well as reviews that focus on nursing education. Finally, scoping reviews are not intended to make direct recommendations for practice, since the main purpose of this specific type of evidence synthesis is to chart the existing available evidence. Therefore, we simply propose areas that the findings may serve as a foundation to inform future work to inform practice change, which include nursing education, clinical care, research, policy, and administration.

## Conclusion

This is the first review that charts evidence regarding Black nurses in Canada, which provides insight and direction for future research, particularly in alignment with the growing necessity for the collection of race-disaggregated data. As outlined in the a priori protocol, the review findings are presented according to five overarching categories, to address the review question and objectives. These categories include historical situatedness; immigration; racism and discrimination; leadership and career progression and finally, diversity in the workforce. The findings of this review capture several critical elements including the pervasiveness of racism as well as the emphasis on policy and the importance of engaging in policy evaluation. The relevance of this review is extremely important for scholars interested and engaged in addressing health inequities and disparities for historically marginalized groups as well as the advancement of the nursing profession and healthcare. Collectively, this review facilitates understanding history in a way that enables forward direction. It is impossible to strive towards equity, diversity, and inclusion in nursing without an honest conversation and understanding about the past, as problematic as it may be. Uncovering the past is not meant to place blame but rather generate an understanding as to what the issues were, challenges that persist and ways to more forward together. The legacy of anti-Black racism in the nursing profession was shown to permeate all sectors of nursing – from clinical care, administration, research, policy, and education. To move beyond the historical legacy of anti-Black racism and discrimination in nursing, it is vital to understand the historical context in relation to Black nurses in addition to strategic planning for an inclusive profession.

### Limitations

Potential limitations for this review include the search strategy, which may have missed sources. It is possible that human error in screening may have unintentionally excluded sources. Moreover, studies that focused on visible minorities, IENs or nursing cohorts that did not include data that were disaggregated by race and/or ethnicity were excluded. It is possible that these sources included data regarding Black nurses in Canada, however, without adequate disaggregation, the data could not be used.

## Appendix 1

### Full search strategies

CINAHL

**Table:** 

	Search Date: August 31^st^, 2020	
**#**	**Query**	**Results**
S9	S5 AND S8	154
S8	S6 OR S7	137,843
S7	TI ( Canad* OR "British Columbia" OR "British Colombian" OR Alberta OR Albertan OR Saskatchewan OR Saskatchewanian OR Manitoba OR Manitoban OR Ontario OR Ontarian OR Quebec OR Quebecer OR Quebecois OR "New Brunswick" OR "New Brunswicker" OR "Nova Scotia" OR "Nova Scotian" OR "Prince Edward Island" OR "Prince Edward Islander" OR Newfoundland OR Newfoundlander OR Labrador OR "Northwest Territories" OR "Northwest Territorian" OR Yukon OR Yukoner OR Nunavut OR Nunavummiut) OR AB ( Canad* OR "British Columbia" OR "British Colombian" OR Alberta OR Albertan OR Saskatchewan OR Saskatchewanian OR Manitoba OR Manitoban OR Ontario OR Ontarian OR Quebec OR Quebecer OR Quebecois OR "New Brunswick" OR "New Brunswicker" OR "Nova Scotia" OR "Nova Scotian" OR "Prince Edward Island" OR "Prince Edward Islander" OR Newfoundland OR Newfoundlander OR Labrador OR "Northwest Territories" OR "Northwest Territorian" OR Yukon OR Yukoner OR Nunavut OR Nunavummiut)	84,317
S6	(MH "Canada + ")	102,452
S5	S3 OR S4	8,815
S4	TI ( (Black OR African OR Afro* OR Coloured OR Colored OR Caribbean OR West Indian OR West Indies OR "of colour" OR "of color" OR minority) AND (nurse OR nursing OR nurses)) OR AB ( (Black OR African OR Coloured OR Colored OR Caribbean OR West Indian OR West Indies "of colour" OR "of color" OR minority) AND (nurse OR nursing OR nurses))	7,756
S3	S1 AND S2	1,820
S2	(MH "Nurses + ")	224,727
S1	(MH "Blacks") OR (MH "Minority Groups")	63,761
	Uploaded to Covidence 147 (7 duplicates removed)	

**Table:** 

#	Searches	Results
1	exp african continental ancestry group/ or african americans/	86940
2	Minority Groups/	13959
3	1 or 2	98483
4	exp Nurses/	88336
5	3 and 4	474
6	((Black or African or Afro* or Coloured or Colored or Caribbean or West Indian or West Indies or "of colour" or "of color" or minority) and (nurse or nursing or nurses)).ti,ab,kw,kf	7438
7	5 or 6	7732
8	exp Canada/	159063
9	(Canad* or "British Columbia" or "British Colombian" or Alberta or Albertan or Saskatchewan or Saskatchewanian or Manitoba or Manitoban or Ontario or Ontarian or Quebec or Quebecer or Quebecois or "New Brunswick" or "New Brunswicker" or "Nova Scotia" or "Nova Scotian" or "Prince Edward Island" or "Prince Edward Islander" or Newfoundland or Newfoundlander or Labrador or "Northwest Territories" or "Northwest Territorian" or Yukon or Yukoner or Nunavut or Nunavummiut).ti,ab,kw,kf	169248
10	8 or 9	238193
11	7 and 10	154
	Uploaded to Covidence 80 (74 duplicates removed)	

Embase.

**Table:** 

No	Query	Results
#9	#5 AND #8	188
#8	#6 OR #7	287456
#7	canad*:ti,ab,kw OR 'british columbia':ti,ab,kw OR 'british colombian':ti,ab,kw OR alberta:ti,ab,kw OR albertan:ti,ab,kw OR saskatchewan:ti,ab,kw OR saskatchewanian:ti,ab,kw OR manitoba:ti,ab,kw OR manitoban:ti,ab,kw OR ontario:ti,ab,kw OR ontarian:ti,ab,kw OR quebec:ti,ab,kw OR quebecer:ti,ab,kw OR quebecois:ti,ab,kw OR 'new brunswick':ti,ab,kw OR 'new brunswicker':ti,ab,kw OR 'nova scotia':ti,ab,kw OR 'nova scotian':ti,ab,kw OR 'prince edward island':ti,ab,kw OR 'prince edward islander':ti,ab,kw OR newfoundland:ti,ab,kw OR newfoundlander:ti,ab,kw OR labrador:ti,ab,kw OR 'northwest territories':ti,ab,kw OR 'northwest territorian':ti,ab,kw OR yukon:ti,ab,kw OR yukoner:ti,ab,kw OR nunavut:ti,ab,kw OR nunavummiut:ti,ab,kw	227083
#6	'canada'/exp	189364
#5	#3 OR #4	9967
#4	(black:ti,ab,kw OR african:ti,ab,kw OR afro* OR coloured:ti,ab,kw OR colored:ti,ab,kw OR caribbean:ti,ab,kw OR 'west indian':ti,ab,kw OR 'west indies':ti,ab,kw OR 'of colour':ti,ab,kw OR 'of color':ti,ab,kw OR minority:ti,ab,kw) AND (nurse:ti,ab,kw OR nursing:ti,ab,kw OR nurses:ti,ab,kw)	9509
#3	#1 OR #2	1250
#2	'nurse'/exp	182676
#1	'black person'/exp OR 'minority group'/exp	145793
	Uploaded to Covidence 69 (119 duplicates removed)	

Sociological abstracts.

**Table:** 

Search	Databases	Actions
**S3**	1 AND 2	**27**
**S2**	ti(Canad* OR "British Columbia" OR "British Colombian" OR Alberta OR Albertan OR Saskatchewan OR Saskatchewanian OR Manitoba OR Manitoban OR Ontario OR Ontarian OR Quebec OR Quebecer OR Quebecois OR "New Brunswick" OR "New Brunswicker" OR "Nova Scotia" OR "Nova Scotian" OR "Prince Edward Island" OR "Prince Edward Islander" OR Newfoundland OR Newfoundlander OR Labrador OR "Northwest Territories" OR "Northwest Territorian" OR Yukon OR Yukoner OR Nunavut OR Nunavummiut) OR ab(Canad* OR "British Columbia" OR "British Colombian" OR Alberta OR Albertan OR Saskatchewan OR Saskatchewanian OR Manitoba OR Manitoban OR Ontario OR Ontarian OR Quebec OR Quebecer OR Quebecois OR "New Brunswick" OR "New Brunswicker" OR "Nova Scotia" OR "Nova Scotian" OR "Prince Edward Island" OR "Prince Edward Islander" OR Newfoundland OR Newfoundlander OR Labrador OR "Northwest Territories" OR "Northwest Territorian" OR Yukon OR Yukoner OR Nunavut OR Nunavummiut)	**50775**
**S1**	ti((Black OR African OR Afro* OR Coloured OR Colored OR Caribbean OR West Indian OR West Indies OR "of colour" OR "of color" OR minority) AND (nurse OR nursing OR nurses)) OR ab((Black OR African OR Afro* OR Coloured OR Colored OR Caribbean OR West Indian OR West Indies OR "of colour" OR "of color" OR minority) AND (nurse OR nursing OR nurses))	**567**
	Uploaded to Covidence 18 (9 duplicates removed)	

Gender studies database.

**Table:** 

#	Query	Results
S3	S1 AND S2	31
S2	TI ( Canad* OR "British Columbia" OR "British Colombian" OR Alberta OR Albertan OR Saskatchewan OR Saskatchewanian OR Manitoba OR Manitoban OR Ontario OR Ontarian OR Quebec OR Quebecer OR Quebecois OR "New Brunswick" OR "New Brunswicker" OR "Nova Scotia" OR "Nova Scotian" OR "Prince Edward Island" OR "Prince Edward Islander" OR Newfoundland OR Newfoundlander OR Labrador OR "Northwest Territories" OR "Northwest Territorian" OR Yukon OR Yukoner OR Nunavut OR Nunavummiut) OR AB ( Canad* OR "British Columbia" OR "British Colombian" OR Alberta OR Albertan OR Saskatchewan OR Saskatchewanian OR Manitoba OR Manitoban OR Ontario OR Ontarian OR Quebec OR Quebecer OR Quebecois OR "New Brunswick" OR "New Brunswicker" OR "Nova Scotia" OR "Nova Scotian" OR "Prince Edward Island" OR "Prince Edward Islander" OR Newfoundland OR Newfoundlander OR Labrador OR "Northwest Territories" OR "Northwest Territorian" OR Yukon OR Yukoner OR Nunavut OR Nunavummiut)	21,030
S1	TI ( (Black OR African OR Afro* OR Coloured OR Colored OR Caribbean OR West Indian OR West Indies OR "of colour" OR "of color" OR minority) AND (nurse OR nursing OR nurses)) OR AB ( (Black OR African OR Coloured OR Colored OR Caribbean OR West Indian OR West Indies "of colour" OR "of color" OR minority) AND (nurse OR nursing OR nurses))	701
	Uploaded to Covidence 18 (13 duplicates removed)	

America: history & life.

**Table:** 

#	Query	Results
S3	S1 AND S2	10
S2	TI ( Canad* OR "British Columbia" OR "British Colombian" OR Alberta OR Albertan OR Saskatchewan OR Saskatchewanian OR Manitoba OR Manitoban OR Ontario OR Ontarian OR Quebec OR Quebecer OR Quebecois OR "New Brunswick" OR "New Brunswicker" OR "Nova Scotia" OR "Nova Scotian" OR "Prince Edward Island" OR "Prince Edward Islander" OR Newfoundland OR Newfoundlander OR Labrador OR "Northwest Territories" OR "Northwest Territorian" OR Yukon OR Yukoner OR Nunavut OR Nunavummiut) OR AB ( Canad* OR "British Columbia" OR "British Colombian" OR Alberta OR Albertan OR Saskatchewan OR Saskatchewanian OR Manitoba OR Manitoban OR Ontario OR Ontarian OR Quebec OR Quebecer OR Quebecois OR "New Brunswick" OR "New Brunswicker" OR "Nova Scotia" OR "Nova Scotian" OR "Prince Edward Island" OR "Prince Edward Islander" OR Newfoundland OR Newfoundlander OR Labrador OR "Northwest Territories" OR "Northwest Territorian" OR Yukon OR Yukoner OR Nunavut OR Nunavummiut)	66810
S1	TI ( (Black OR African OR Afro* OR Coloured OR Colored OR Caribbean OR West Indian OR West Indies OR "of colour" OR "of color" OR minority) AND (nurse OR nursing OR nurses)) OR AB ( (Black OR African OR Coloured OR Colored OR Caribbean OR West Indian OR West Indies "of colour" OR "of color" OR minority) AND (nurse OR nursing OR nurses))	124
	Uploaded to Covidence 5 (5 duplicates removed)	

PsycINFO.

**Table:** 

#	Query	Results
S8	S2 AND S7	45
S7	S1 OR S6	2,848
S6	S4 AND S5	173
S5	DE "Nurses" OR DE "Psychiatric Nurses" OR DE "Public Health Service Nurses" OR DE "School Nurses"	32,449
S4	(DE "Blacks") OR (DE "Minority Groups")	67,326
S3	S1 AND S2	45
S2	TI ( Canad* OR "British Columbia" OR "British Colombian" OR Alberta OR Albertan OR Saskatchewan OR Saskatchewanian OR Manitoba OR Manitoban OR Ontario OR Ontarian OR Quebec OR Quebecer OR Quebecois OR "New Brunswick" OR "New Brunswicker" OR "Nova Scotia" OR "Nova Scotian" OR "Prince Edward Island" OR "Prince Edward Islander" OR Newfoundland OR Newfoundlander OR Labrador OR "Northwest Territories" OR "Northwest Territorian" OR Yukon OR Yukoner OR Nunavut OR Nunavummiut) OR AB ( Canad* OR "British Columbia" OR "British Colombian" OR Alberta OR Albertan OR Saskatchewan OR Saskatchewanian OR Manitoba OR Manitoban OR Ontario OR Ontarian OR Quebec OR Quebecer OR Quebecois OR "New Brunswick" OR "New Brunswicker" OR "Nova Scotia" OR "Nova Scotian" OR "Prince Edward Island" OR "Prince Edward Islander" OR Newfoundland OR Newfoundlander OR Labrador OR "Northwest Territories" OR "Northwest Territorian" OR Yukon OR Yukoner OR Nunavut OR Nunavummiut)	54,759
S1	TI ( (Black OR African OR Afro* OR Coloured OR Colored OR Caribbean OR West Indian OR West Indies OR "of colour" OR "of color" OR minority) AND (nurse OR nursing OR nurses)) OR AB ( (Black OR African OR Coloured OR Colored OR Caribbean OR West Indian OR West Indies "of colour" OR "of color" OR minority) AND (nurse OR nursing OR nurses))	2,821
	Uploaded to Covidence 17 (28 duplicates removed)	

Academic search premier.

**Table:** 

#	Query	Results
S3	S1 AND S2	38
S2	TI ( Canad* OR "British Columbia" OR "British Colombian" OR Alberta OR Albertan OR Saskatchewan OR Saskatchewanian OR Manitoba OR Manitoban OR Ontario OR Ontarian OR Quebec OR Quebecer OR Quebecois OR "New Brunswick" OR "New Brunswicker" OR "Nova Scotia" OR "Nova Scotian" OR "Prince Edward Island" OR "Prince Edward Islander" OR Newfoundland OR Newfoundlander OR Labrador OR "Northwest Territories" OR "Northwest Territorian" OR Yukon OR Yukoner OR Nunavut OR Nunavummiut) OR AB ( Canad* OR "British Columbia" OR "British Colombian" OR Alberta OR Albertan OR Saskatchewan OR Saskatchewanian OR Manitoba OR Manitoban OR Ontario OR Ontarian OR Quebec OR Quebecer OR Quebecois OR "New Brunswick" OR "New Brunswicker" OR "Nova Scotia" OR "Nova Scotian" OR "Prince Edward Island" OR "Prince Edward Islander" OR Newfoundland OR Newfoundlander OR Labrador OR "Northwest Territories" OR "Northwest Territorian" OR Yukon OR Yukoner OR Nunavut OR Nunavummiut)	480,039
S1	TI ( (Black OR African OR Afro* OR Coloured OR Colored OR Caribbean OR West Indian OR West Indies OR "of colour" OR "of color" OR minority) N4 (nurse OR nursing OR nurses)) OR AB ( (Black OR African OR Coloured OR Colored OR Caribbean OR West Indian OR West Indies "of colour" OR "of color" OR minority) N4 (nurse OR nursing OR nurses))	1,527
	Uploaded to Covidence 13 (25 duplicates removed)	

Scopus.

(TITLE-ABS-KEY((Black OR African OR Afro* OR Coloured OR Colored OR Caribbean OR "West Indian" OR "West Indies" OR "of colour" OR "of color" OR minority) W/4 (nurse OR nursing OR nurses))) AND ((TITLE-ABS-KEY(Canad* OR "British Columbia" OR "British Colombian" OR Alberta OR Albertan OR Saskatchewan OR Saskatchewanian OR Manitoba OR Manitoban OR Ontario OR Ontarian OR Quebec OR Quebecer OR Quebecois OR "New Brunswick" OR "New Brunswicker" OR "Nova Scotia")) OR (TITLE-ABS-KEY("Nova Scotian" OR "Prince Edward Island" OR "Prince Edward Islander" OR Newfoundland OR Newfoundlander OR Labrador OR "Northwest Territories" OR "Northwest Territorian" OR Yukon OR Yukoner OR Nunavut OR Nunavummiut))).

48 results

## Appendix 2

### Articles excluded at full-text – with rationale

#### Context issue (not specific to canada)

1. Cassiani SHDB, Lecorps K, Rojas Canaveral LK, da Silva FAM, Fitzgerald J. Regulation of nursing practice in the Region of the Americas. Revista panamericana de salud publica = Pan American journal of public health. 2020;44(csl, 9705400):e93.
2. D’Antonio P. Thinking about place: Researching and reading the global history of nursing. Texto Contexto Enferm. 2009;18(4):766–72.
3. Grypma SJ. Leadership in history. Profile of a leader: unearthing Ethel Johns’s “buried” commitment to racial equality, 1925. Nurs Leadersh 2003,16(4):39–47.
4. Jefferies K. A Personal Leadership Development Plan for Black Undergraduate and Graduate Nursing Students. Nurs leadersh (Tor ONT)0.2018, 31(4):57–62.
5. Jefferies K. The Strong Black Woman: Insights and Implications for Nursing. J Am Psychiatr Nurs Assoc. 2020.
6. Jefferies K, Goldberg L, Aston M, Tomblin Murphy G. Understanding the invisibility of black nurse leaders using a black feminist poststructuralist framework. J Clin Nurs (John Wiley & Sons, Inc). 2018;27(15–16):3225–34
7. Johnson SA. Healing in silence: black nurses in Charleston, South Carolina, 1896–1948. Medical University of South Carolina 2008.
8. Kawi J, Xu Y. Facilitators and barriers to adjustment of international nurses: An integrative review. Int Nurs Rev. 2009,56(2):174–83.
9. Lewenson SB, Graham-Perel A. You don’t have any business being this good: An oral history interview with Bernardine Lacey. Am J Nurs. 2020;120(8):40–7
10. Likupe G. The skills and brain drain what nurses say. J Clin Nurs (John Wiley & Sons, Inc). 2013;22(9–10):1372–81
11. Ocho ON, Wheeler E, Sheppard C, Caesar-Greasley L-A, Rigby J, Tomblin Murphy G. Nurses’ preparation for transitioning into positions of leadership—A Caribbean perspective. J Nurs Manage. 2020,28(6):1356–63.
12. Ohr SO, Jeong S, Parker V, McMillan M. Organizational support in the recruitment and transition of overseas-qualified nurses: Lessons learnt from a study tour. Nurs Health Sci. 2014;16(2):255–61
13. Xu Y. Racism and discrimination in nursing: Reflections on multicultural nursing conference. Home Health Care Manag Pract. 2008;20(3):284–6.
14. Young J. Revisiting the 1925 Johns Report on African-American nurses. Nurs Hist Rev. 2005;13(bqa, 9303945):77–99

#### Population issue (not specific to black nurses in Canada)

1. Agnew V, Hagey R, Turrittin J, Das Gupta T. Racial discrimination in nursing. In: Interrogating Race and Racism. Toronto, Ontario: University of Toronto Press; 2007. 206–36 p.
2. Bell B. White dominance in nursing education: A target for anti-racist efforts. Nurs Inq [Internet]. 2021;28(1). Available from: https://www.scopus.com/inward/record.uri?eid=2-s2.0-85090159403&doi=10.1111%2fnin.12379&partnerID=40&md5=9e5527db95846cbec584b607282c5c26
3. Blanchet Garneau A, Browne AJ, Varcoe C. Drawing on antiracist approaches toward a critical antidiscriminatory pedagogy for nursing. Nurs Inq.2018,25(1).
4. Blythe J, Baumann A. Internationally educated nurses: Profiling workforce diversity. Int Nurs Rev. 2009,56(2):191–7.
5. Choiniere JA, MacDonnell J, Shamonda H. Walking the talk: Insights into dynamics of race and gender for nurses. Policy Polit Nurs Pract. 2010;11(4):317–25
6. Covell CL, Neiterman E, Bourgeault IL. Scoping review about the professional integration of internationally educated health professionals. Hum Resour Health. 2016;14(1).
7. Covell CL, Rolle Sands S. Does Being a Visible Minority Matter? Predictors of Internationally Educated Nurses’ Workplace Integration. Can J Nurs Res. 2020.
8. Creese G, Stasiulis D. Introduction: Intersections of Gender, Race, Class, and Sexuality. Stud Polit Econ.1996, (51):5–14.
9. Duncan S, Whyte N. Global leadership priorities for Canadian nursing: a perspective on the ICN 24th Quadrennial Congress, Durban, South Africa. Nurs leadersh (Tor ONT). 2010,23(1):16–21.
10. Dyck I. Negotiating citizenship: migrant women in Canada and the global system. Fem Rev.2004, (77):201–3.
11. English HA. The journals of Helen Anne English, field Matron on the Little Pine Reserve, 1913–1917. Saskatchewan History Magazine. 1993;45(2):37–42
12. Etowa JB, Foster S, Vukic AR, Wittstock L, Youden S. Recruitment and retention of minority students: Diversity in nursing education. Int J Nurs Educ Scholarsh. 2005;2(1):12p–12p
13. Glasgow VM. Race and employment equity in nursing leadership: Perceptions of racialized and non-racialized registered nurses [Internet]. ProQuest Information & Learning; 2019.
14. Goodwin JO. Improving the recruitment and retention of registered nurses through the identification of critical success factors: A comparative study of the United States and Canada. Central Michigan University.2010.
15. Guruge S, Donner G. Transcultural nursing in Canada. Can Nurse. 1996 Sep;92(8):36–40
16. Hussain H, McGeer A, McNeil S, Katz K, Loeb M, Simor A, et al. Factors associated with influenza vaccination among healthcare workers in acute care hospitals in Canada. Influenza other Respir viruses. 2018,12(3):319–25.
17. Jefferies K, Tamlyn D, Aston M, Tomblin Murphy G. Promoting Visible Minority Diversity in Canadian nursing. Can J Nurs Res. 2019;51(1):3–5
18. Knight M. Friends of African nursing (Canada). Can Oper Room Nurs J. 2011, 29(1):16–20.
19. Labun E. The Red River College model: Enhancing success for Native Canadian and other nursing students from disenfranchised groups. J Transcult Nurs. 2002;13(4):311–7.
20. Lane J, Carrier L, Jefferies K, Yu Z. Diverse Representation in Nursing Leadership: Developing a Shared Position Statement on Allyship. Creat Nurs. 2019,25(4):316–21.
21. MacDonnell JA. Situating the political in nurses’ lives: The intersection of policy, practice and career for lesbian health advocates. University of Toronto (Canada); 2005.
22. Mansell D, Hibberd J. Forcible sterilization in Canada, 1920–1940. “We picked the wrong one to sterilise”: the role of nursing in the eugenics movement in Alberta, 1920–1940. Inter Hist Nurs J. 1998, 3(4):4–11.
23. Mansell D, Hibberd J. “We picked the wrong one to sterilise”: The role of nursing in the eugenics movement in Alberta, 1920–1940. Int Hist Nurs J. 1998;3(4):4–11
24. Maree J, Fitch MI. Holding conversations with cancer patients about sexuality: Perspectives from Canadian and African healthcare professionals. Can Oncol Nurs J. 2019,29(1):64–76.
25. McPherson K. Bedside matters: The transformation of Canadian nursing, 1900–1990. The Transformation of Canadian Nurs. 1900–1990. 2003.
26. Meyer M, Estable AR, MacLean L, Peterson WE. Family Home Visitors: Increasing Minority Women’s Access to Health Services. J Health Dispar Res Prac. 2010,3(3):1–20.
27. Mill J, Davison C, Richter S, Etowa J, Edwards N, Kahwa E, et al. Qualitative research in an international research program: Maintaining momentum while building capacity in nurses. Int J Qual Methods. 2014;13(1):151–69
28. Moyce S, Lash R, de Leon Siantz ML. Migration Experiences of Foreign Educated Nurses: A Systematic Review of the Literature. J Transcult Nurs. 2016,27(2):181–8.
29. Moynagh M. Writing Black Canadas. Can Lit. 2008;(198):158–9
30. Neiterman E, Bourgeault IL. The shield of professional status: Comparing internationally educated nurses’ and international medical graduates’ experiences of discrimination. Health. 2015;19(6):615–34
31. Neiterman E, Bourgeault IL. Professional integration as a process of professional resocialization: Internationally educated health professionals in Canada. Soc Sci Med. 2015;131:74–81.
32. Nevitt J, Tunis B. White caps and black bands: Nursing in Newfoundland to 1934 (1978). Atlantis. 1980;5(2):235–9
33. Njie-Mokonya N. Internationally educated nurses’ and their contributions to the patient experience. Online J Issues Nurs. 2016,21(1).
34. Nourpanah S. “Maybe we shouldn’t laugh so loud”: The hostility and welcome experienced by foreign nurses on temporary work permits in Nova Scotia, Canada. Labour Travail. 2019, (83):105–20.
35. O’Brien-Pallas L, Wang S. Innovations in health care delivery: responses to global nurse migration–a research example. Policy Polit Nurs Pract. 2006,7(3S):49S-57S.
36. Omene M. A Study of the Experiences of Black Caribbean Women in the Saskatchewan Labour Force. Masters Abstr Int 0.2001,39(03):733–733.
37. Oosterbroek T, Ponomar V. “It All Depends”: How Minority Nursing Students Experience Belonging During Clinical Experiences. Nurs Educ Perspect.2014,35(2):89–93.
38. Pittman P, Aiken LH, Buchan J. International migration of nurses: Introduction. Health Services Research. 2007,42(3, part2):1275–80.
39. Prentice D, Moore J, Crawford J, Lankshear S, Limoges J. Collaboration among Registered Nurses and Licensed Practical Nurses: A Scoping Review of Practice Guidelines. Nurs Res Pract 0.2020.
40. Ramji Z, Etowa J. Towards a conceptual framework for workplace integration of internationally educated nurses. Manage Educ Int J. 2015;15(3):1–11
41. Rasmussen BM, Marie Garran A. In the Line of Duty: Racism in Health Care. Soc Work. 2016,61(2):175–7.
42. Richmond AH. Caribbean immigrants in Britain and Canada: socio-economic adjustment. Int Migr.1988,26(4):365–86.
43. Runnels V, Labonté R, Packer C. Reflections on the ethics of recruiting foreign-trained human resources for health. Hum Resour Health. 2011;9
44. Rutherdale M. Introduction. Caregiving on the Peripher: Hist Perspect on Nurs and Midwifery in Can. 2010;3–32.
45. Spitzer DL. In visible bodies: minority women, nurses, time, and the new economy of care. Med Anthropol Q. 2004,18(4):490–508.
46. Tastsoglou E, Miedema B. “Working Much Harder and Always Having to Prove Yourself”: Immigrant Women's Labor Force Experiences in the Canadian Maritimes. Adv Gender Res. 9:201–33.
47. Timer JE, Clauson MI. The use of selective admissions tools to predict students’ success in an advanced standing baccalaureate nursing program. Nurse Educ Today. 2011;31(6):601–6
48. Tomblin Murphy G, Birch S, MacKenzie A, Alder R, Lethbridge L, Little L. Eliminating the shortage of registered nurses in Canada: An exercise in applied needs-based planning. Health Policy. 2012,105(2–3):192–202.
49. Tuttas CA. Perceived racial and ethnic prejudice and discrimination experiences of minority migrant nurses: A literature review. J Transcult Nurs. 2015;26(5):514–20.
50. Twomey JC, Meadus R. Men nurses in Atlantic Canada: Career choice, barriers, and satisfaction. J Mens Stud. 2016,24(1):78–88.
51. Varcoe C, McCormick J. Racing around the classroom margins: Race, racism, and teaching nursing. In: Teach Nurs: Dev A Stud-Centered Learn Environ. 2012. 437–66 p.
52. Walani SR. Global migration of internationally educated nurses: Experiences of employment discrimination. Int J Afr Nurs Sci. 2015, 3:65–70.
53. Wong S, Wong J. Representation of racial minority students in selected Canadian university schools of nursing. J Adv Nurs (Wiley-Blackwell). 1980;5(1):83–90

#### Concept issue (not specific to nursing)

1. Maiter S. Using an anti-racist framework for assessment and intervention in clinical practice with families from diverse ethno-racial backgrounds. Clin Soc Work J. 2009;37(4):267–76.
2. Nurse DB, McNeil D. What’s a black critic to do? Interviews, profiles and reviews of black writers. Can Ethn Stud. 2004;36(2):162–4.
3. Sztainbok V. Presumed incompetent: The intersections of race and class for women in academia. Resources for Feminist Research. 2016;34(3/4):157–161,164.
4. THOMPSON C. Cultivating Narratives of Race, Faith, and Community: The Dawn of Tomorrow, 1923–1971. Can J Hist 2015,50(1):30–67.

#### Other category: (book review)

1. Bates C, Dodd D, Rousseau N. On all frontiers: Four centuries of Canadian nursing. University of Ottawa Press. 2005.
2. Butler A. Moving Beyond Borders: A History of Black Canadian and Caribbean Women in the Diaspora. Can Ethn Stud 2013,45(3):159–61.
3. Fingard J, Guildford JV. Mothers of the municipality: Women, work and social policy in post-1945 Halifax. 2005.
4. Foth T. Moving Beyond Borders: A History of Black Canadian and Caribbean Women in the Diaspora. Nurs Hist Rev. 2013,21:151–2.
5. Wyche KF. Review of Moving beyond borders: A history of Black Canadian and Caribbean women in the diaspora. Affilia: Journal of Women & Social Work. 2013;28(4):475–6.

## Appendix 3

### Categories in Nursing (Included Sources): Research Studies and Non-research Sources

#### Categories in Nursing – Research studies (*n* = 18)

**Table:** 

Author (Year of Publication)	Research Aim/ Purpose	Methodology/ Design	Methods	Study Location	Nursing Concept
Boateng (2015) [28]	To explore the career pathways and experiences of immigrant and Canadian-born nurses in two Ontario cities	Qualitative Research *DISSERTATION	Interviews	Ontario [Toronto and London]	Leadership and Career Advancement
Bouabdillah et al. (2016) [31]	To explore the perspectives of visible minority nurses in relation to their career paths	Qualitative Research Critical Ethnography	Interviews, observations, field notes	Ontario/ Ottawa	Leadership and Career advancement
Calliste (1996) [33]	To examine experiences of women of colour, specifically African Canadian nurses, organizing and resisting racism in nursing in Ontario and Quebec from the late 1970s to the 1990s, from an integrative anti-racism perspective	Qualitative Research Integrative Anti-Racism	Interviews	Ontario and Quebec	Racism
Calliste (1993) [32]	To examine Canada's immigration policy on Caribbean nurses and nursing assistants during the post-war industrial and baby boom period, 1950 to1962	Qualitative Research	Document review, Literature Search & Interviews	National	Immigration/ Migration
Collins (2004) [34]	To investigate the experiences of immigrant women from the Caribbean who are registered nurses (RNs) in Canada	Qualitative Research Descriptive *DISSERTATION	Interviews	Ontario	Leadership and Career Advancement
Das Gupta (2009) [37]	To develop a theoretical framework for understanding systemic racism	Mixed Methods Research *BOOK	Literature review, surveys	Ontario	Racism
Das Gupta (1996) [36]	To describe the experience of racism in nursing in Ontario drawing on the case histories of two Black nurses who have brought complaints against their hospital to the OHRC. [Included this experience of racism in light of experiences documented in other sources.]	Qualitative Research	Interviews & case review	Ontario	Racism
Etowa (2005) [38]	To discover the nature of work life experiences of Black nurses in the health care system in Nova Scotia	Qualitative Research Grounded Theory *DISSERTATION	Interviews, literature review, field notes, observation, group discussion	Nova Scotia	Worklife/ Diversity in Nursing
Flynn (2011) [46]	To describe the lives of Caribbean and Canadian born Black professional women	Qualitative Research *BOOK	Interviews	Ontario	History
Hagey et al. (2001) [51]	To document and describe the experiences of immigrant nurses of colour who have filed grievances concerning their employers’ discriminatory practices; and to solicit their views of existing policies and recommendations for equity in professional life	Qualitative Research Analytical Framework: Everyday Racism and Discourse Analysis	Interviews	Ontario	Racism
Keddy (1997) [53]	To recover identities of Black nurses to help shed [light on] social issues that shape the profession today	Qualitative Research	Interviews (Oral Histories)	Nova Scotia	History
Labonté et al. (2006) [54]	To ascertain recent trends on health human resource (HHR) flows, perceived reasons for such flows, and key Canadian stakeholder awareness of, and support for, options by which Canada might help mitigate the negative effects of HHR migration from this region	Mixed Method	Interviews and Secondary Analysis	National	Immigration/ Migration
Modibo (2004) [56]	To present the everyday workplace experiences of racism that African Canadian nurses confronted in some of Toronto's hospitals in the decade that followed the letter's receipt	Qualitative Research	Interviews	Ontario [Toronto]	Racism
Premji et al. (2014) [57]	To develop a diversity profile of the nursing workforce in Canada and its major cities	Quantitative Research	Secondary Analysis	National/ Multi-city [Toronto, Vancouver, Montreal, Halifax]	Diversity in Nursing
Prendergast (2014) [58]	To examine the roles of the ideal type and multiculturalism policies within nursing and questions whether it works in favour of internationally educated nurses of colour or more as a hindrance to their educational and promotional development	Qualitative Research Post-colonial, anti-racist feminist and Black Canadian feminist theory *DISSERTATION	Interviews	Ontario	Leadership and Career Advancement
Racine (2009) [59]	To present experiences of everyday racism observed and collected in a critical ethnography among a group of Haitian Canadians in Quebec	Qualitative Research Post-colonial Feminist perspective/ Critical Ethnography	Interviews	Quebec	Racism
Sands et al. (2020) [60]	To examine the amount, type, sources, distribution, and focus of the conceptual and empirical literature on migration of Caribbean nurses and to identify gaps in the literature	Scoping Review 5 stage framework	Review of Literature	National	Immigration/ Migration
Stewart (2009) [62]	To examines the impact of race on the workplace experiences of Black women in nursing leadership positions	Qualitative Research Integrative anti-racism and Black feminism *DISSERTATION	Interviews	Ontario [Toronto]	Leadership and Career Advancement

#### Categories in Nursing – Non-research sources (*n* = 13)

**Table:** 

Author (Year)	Source Title	Source Type	Concept in Nursing
Congress of Black Women Canada—Toronto Chapter (1995) [35]	End the silence on racism in health care: Build a movement against discrimination, harassment and reprisals	Commentary	Racism
Canadian Nurses Association (2020) [65]	CNA’s Key Messages on Anti-Black Racism in Nursing and Health	Key Messages	Racism
Flynn (2019) [50]	Writing Black Canadian Women's History: Where We Have Been and Where We are Going	Commentary	Racism
Flynn (2018) [49]	"Hotel Refuses Negro Nurse ": Gloria Clarke Baylis and the Queen Elizabeth Hotel	Commentary	Racism
Jefferies (2020) [66]	Recognizing history of Black nurses: a first step to addressing racism and discrimination in nursing	Commentary	Racism
Jefferies et al. (2018) [52]	Black Nurse Leaders in the Canadian Healthcare System	Commentary	Leadership
Missen (2010) [55]	De l'Afrique a Winnipeg: Three Nursing Journeys	Commentary	Immigration
Registered Nurses Association of Ontario (RNAO) (2020^a^) [67]	RNAO stands together with our black sisters and brothers	Announcement	Racism
Registered Nurses Association of Ontario (RNAO) (2020^b^) [68]	RNAO stands together with our black sisters and brothers	Media Statement	Racism
Registered Nurses Association of Ontario (RNAO) (2015) [69]	A History of Diversity & Inclusivity: In celebration of RNAO’s 90^th^ anniversary – and to mark February as Black History Month – we take a look back at the association’s work on diversity, a matter that has shaped the profession from the mid-twentieth century to today	Report	Diversity
Registered Nurses Association of Ontario (RNAO) (2011) [70]	RNs Mark the passing of a true leader	Memorandum	Leadership
Registered Nurses Association of Ontario (RNAO) (2002) [71]	Policy Statement: Racism	Policy Statement	Racism
Villeneuve (2003) [64]	Healthcare, Race and Diversity: Time to Act	Commentary	Diversity

## Appendix 4

### Description of included studies (primary source)

**Table:** 

Authors/ Year	Title	Design/ Method	Aim/ Purpose	Concept	Province/ City	African Canadian Terminology	Gender & Sample	Key Findings
Boateng, G. 2015 [32]	Exploring the Career Pathways, Professional Integration and Lived Experiences of Regulated Nurses in Ontario, Canada	Qualitative Research Interviews Dissertation	This study explores the career pathways and experiences of immigrant and Canadian-born nurses in two Ontario cities utilizing a qualitative research design consisting of 70 in-depth interviews	Leadership and career advancement	Ontario/ Toronto and London	Ethnic minority/ Black/	70 RNs and RPNs 42 immigrant nurses 15 IENs	Canadian-born nurses have a shorter, more direct pathway to nursing. IENs and VMs face systemic issues. VMs experience verbal abuse
Bouabdillah et al. 2016 [35]	Infirmières issues de minorités visibles et mobilité vertical en milieu hospitalier [Visible minority nurses and vertical mobility in hospitals]	Qualitative Research Postcolonical approaches/ Critical Ethnography Interviews, observations, field notes	To explore the perspectives of visible minority nurses in relation to their career paths	Leadership and career advancement	Ontario/ Ottawa	Immigrant visible minority	8 visible minority nurses 1st or 2nd generation immigrant	Barriers kept nurses at lower levels in institutional/ nursing hierarchy, including discriminatory hiring and promotion process
Canadian Nurses Association (CNA). 2021 [26]	CNA’s key messages on anti-Black racism in nursing and health	Key Messages	CNA supports the call for enhanced collection and analysis of race and ethnicity data in partnership with racialized communities. We further call for collaborative structures to ensure identified health disparities are addressed	Anti-Black racism in nursing	National	Black	N/A	Anti-Black racism as a part of Canadian nursing [history]
Calliste, A. 1996 [36]	Antiracism Organizing and Resistance in Nursing: African Canadian Women*	Qualitative Research Integrative anti-racism Interviews	This study examines women of color, specifically African Canadian nurses, organizing and resisting racism in nursing in Ontario and Quebec from the late 1970s to the 1990s, from an integrative anti-racism perspective	Racism in Nursing	Ontario and Quebec	African Canadian / Black	Women 22 RNs	Racially specific gender and classist ideologies reinforce the racial division of labour, exploitation and devaluation of black women's labour. Economic restructuring has disproportionate impact on black nurses
Calliste, A. 1993 [37]	Women of 'Exceptional Merit': Immigration of Caribbean Nurses to Canada	Qualitative Research Document review, Literature Search & Interviews	This study examines Canada's immigration policy on Caribbean nurses and nursing assistants during the post-war industrial and baby boom period, 1950 to1962	Immigration policies	National	Caribbean / Black	Caribbean women	Canadian immigration policy restricted the entry of professional and skilled Caribbean workers. Demonstrates how immigration was controlled by race, class, and gender
Collins, E. 2004 [38]	Career mobility among immigrant registered nurses in Canada: Experiences of Caribbean women	Qualitative Research Descriptive Interviews *Dissertation	This qualitative research study investigated the experiences of immigrant women from the Caribbean who are registered nurses (RNs) in Canada	Leadership and career advancement	Ontario/ Toronto	Caribbean	Women 14 Canadian-Caribbean RNs	Nurses were excluded from opportunities for upward and lateral career mobility, decision making
Congress of Black Women Canada—Toronto Chapter. 1995	End the silence on racism in health care: Build a movement against discrimination, harassment and reprisals	Commentary/ Announcement	Announcement regarding the presentation of their report which included 63 recommendations, themes and initiatives	Anti-Black racism in nursing	Ontario/ Toronto	Black	Black nurses and healthcare workers	Black nurses and other health care workers unfairly dismissed from their jobs, work excessive overtime and have no support in the workplace
Das, Gupta, T. 1996 [40]	Anti-Black Racism in Nursing in Ontario	Qualitative Research/ Interviews & case review	This article describes the experience of racism in nursing in Ontario drawing on the case histories of two Black nurses who have brought complaints against their hospital to the OHRC	Racism in nursing	Ontario	Black	Women 2 Black nurses	Nurses experienced racism that was gendered and classed. Racism operated at various levels including everyday life, at work and attitudes of management and workers
Das Gupta, T. 2009 [41]	Real nurses and Others: Racism in Nursing	Mixed Methods Research Exploratory	The initial objective was to lay bare the common experiences, patterns, features and surface manifestations of systemic racism in nursing in Ontario	Racism in Nursing	Ontario	African/ Black Canadian	Female	The development of a theoretical framework for understanding systematic racism in racism
Etowa, J. 2005 [42]	Surviving on the Margin of a Profession: Experiences of Black Nurses	Qualitative Research Grounded Theory Interviews, literature review, field notes, group discussion, observation *Dissertation	This study sought to discover the nature of work life experiences of Black nurses in the health care system in Nova Scotia	Work-life experiences	Nova Scotia Halifax	Black	20 RNs 3 men 17 women *14ANSs	Although Black nurses are very much insiders by virtue of their professional education, nursing values and culture, they often see themselves practicing outside the center
Flynn, K. / 2018 [53]	"Hotel Refuses Negro Nurse ": Gloria Clarke Baylis and the Queen Elizabeth Hotel	Commentary Intersectionality	Drawing on excerpts from the court transcript, this article expands and complicates intersectionality as a theoretical framework to include other markers of difference. This article focuses on Gloria’s role in the lawsuit	Discrimination Lawsuit	Quebec / Montreal	British-trained Caribbean nurse	1 RN Woman	Chronicling the struggle to integrate into Canadian nursing. Without knowledge of nursing’s exclusionary history, Gloria’s experience could easily be interpreted as an isolated occurrence
Flynn, K./ 2011 [50]	Moving Beyond Borders: A History of Black Canadian and Caribbean Women in the Diaspora	Qualitative Research Interviews * BOOK	The lives of Caribbean and Canadian born Black professional women are the central focus of this research	Experiences navigating education, training, paid and unpaid work	Ontario (mainly) Manitoba, Nova Scotia	Black And Caribbean Canadian Nurses	35 Black women (nurses). 13 born in Canada and 22 born in Caribbean	
Hagey, R. et al. 2001 [55]	Immigrant Nurses’ Experiences of Racism	Qualitative Research/ Analytical framework: Everyday racism and Discourse Analysis Interviews	To document and describe the experiences of immigrant nurses of colour who have filed grievances concerning their employers’ discriminatory practices; and to solicit views of existing policies and recommendations for equity in professional life	Racism	Ontario	Immigrant women of colour	Female 9 immigrant nurses of color	All nurses interviewed experienced reprisals as a result of complaining or filing grievances. Unfairness encountered in the redress process
Jefferies, K. et al. 2018 [21]	Black Nurse Leaders in the Canadian Healthcare System	Commentary	This article highlights a growing gap in the Canadian nursing workforce, specifically in nursing leadership. Black nurses are significantly underrepresented in nursing and even more so as nurse leaders	Leadership	National	Black	Not applicable	Facilitating viability and representation of Black nurse leaders Drawing on their experiential knowledge, Black nurse leaders are able to assist in the development of policies, practice standards and health system reform to better serve the Black community
Jefferies, K. 2020 [86]	Recognizing history of Black nurses a first step to addressing racism and discrimination in nursing	Commentary	Canada’s history of racism and segregation has contributed to residual anti-Black racism that remains present in Canadian nursing	Racism in nursing	National	Black	Not applicable	Nursing can learn from bold, innovative ideas and work towards adopting anti-racist frameworks in education and practice. This begins by actively recognizing, appreciating and celebrating Black nurses and their contributions in nursing
Keddy, B. 1997 [57]	Portrait of Leadership: Stories Shed New Light on Nursing History	Qualitative Research Interviews (Oral Histories)	The use of oral histories to recover identities of Black nurses can help shed [light on] social issues that shape the profession today	Black nurses and nursing history	Nova Scotia	Black	Female 5 Black nurses (3 ANSs, 1 African, 1 Caribbean)	Nurses who spent their childhood in the province (NS) spoke of overt, systemic racism; and, had limited access to Schools- not accepted to program (photo sent with application)
Labonté, R. et al. 2006 [58]	Managing health professional migration from sub-Saharan Africa to Canada: a stakeholder inquiry into policy options	Mixed methods Interviews and Secondary Analysis	We conducted a study to ascertain recent trends on health human resource (HHR) flows, perceived reasons for such flows, and key Canadian stakeholder awareness of, and support for, options by which Canada might help mitigate the negative effects of HHR migration from this region	Migration of nurses from sub-Saharan Africa	National	Sub-Saharan African (SSA)	N/A	Sub-Saharan Africa is not presently a significant source of nurses to Canada, but trends demonstrating a slow but steady increase
Missen, B. 2010 [59]	De l'Afrique a Winnipeg: Three Nursing Journeys	Commentary	To tell the stories of three Franco-Africans who chose to pursue a new life and career in a new land	Immigration	Manitoba, Winnipeg	Franco-Africans	3 nurses (2 women, 1 man)	
	The shattered dreams of African Canadian nurses	Qualitative Research Interviews	To present the everyday workplace experiences of racism that African Canadian nurses confronted in some of Toronto's hospitals in the decade that followed the letter's receipt	Racism	Toronto, Ontario	African Canadian	Female 15 African Canadian nurses	The issues of mistreatment compared to that of their White counterparts in the workplace and verbal abuse by patients for Black nurses
Modibo, N./2004Premji, S. & Etowa, E. 2014 [61]	Workforce utilization of visible and linguistic minorities in Canadian nursing	Quantitative Research Secondary Analysis	This study seeks to develop a diversity profile of the nursing workforce in Canada and its major cities	Diversity profile in the nursing workforce in Canada	National/ Multi-city [Toronto, Vancouver, Montreal, Halifax]	Visible and linguistic minority	Male and female	Provides a diversity profile of the nursing workforce for Canada and its major cities. VMN over-represented in lower-level frontline positions
Prendergast, N. 2014	Multiculturalism Policies: Identifying the dialectic of the “ideal type” within the practices of Canadian nursing	Qualitative Research Theoretical framework: Post-colonial; Anti-racist feminist; Black Canadian feminist *Dissertation	This research examines the roles of the ideal type and multiculturalism policies within nursing and questions whether it works in favour of IENs of colour or more as a hindrance to their educational and promotional development	Leadership	Ontario	Internationally educated nurses (IENs) of colour living in Canada	Female 10 RNs (IENs of color)	Findings exposed multiculturalist ideology was not as useful as initially thought. Relationship between the ideal type and multiculturalism policies
Racine, L. 2009 [63]	Haitian Canadians' Experiences of Racism in Quebec: A Postcolonial Feminist Perspective	Qualitative Research Post-colonial feminist framework Critical Ethnography Interviews	This chapter presents experiences of everyday racism observed and collected in a critical ethnography among a group of Haitian Canadians in Quebec	Racism	Quebec	Haitian Canadians	4 homecare nurses	Experiences of racism in the workplace from patients and colleagues. Discrimination present in hiring practices
Registered Nurses Association of Ontario (RNAO) (2020^a^) [67]	RNAO stands together with our black sisters and brothers	Announcement	The launch of a Black nurses’ task force to tackle anti-Black racism in nursing in response to the marches and rallies organized to honor the life of George Floyd and the many other Black lives that matter	Racism	Ontario	Black	Not applicable	RNAO recognizes that racism is systemic in Canadian society and endemic in institutions
Registered Nurses Association of Ontario (RNAO) (2020^b^) [68]	RNAO stands together with our black sisters and brothers	Media Statement	Express solidarity with those who suffer at the hands of law enforcement and those who experience gross inequities because of their skin color	Racism	Ontario	Black	Not applicable	RNAO has had 3 Black past presidents Follow RNAO online for more information and updates
Registered Nurses Association of Ontario (RNAO) (2015) [69]	A History of Diversity & Inclusivity: In celebration of RNAO’s 90^th^ anniversary – and to mark February as Black History Month – we take a look back at the association’s work on diversity, a matter that has shaped the profession from the mid-twentieth century to today	Report		Diversity	Ontario	Black	Not applicable	Importance of diversity and inclusivity in all of RNAO’s work
Registered Nurses Association of Ontario (RNAO) (2011) [70]	RNs Mark the passing of a true leader	Memorandum	Remembering a nurse leader	Leadership	Ontario	Black	Ms. Lesmond’s decorated career	
Registered Nurses Association of Ontario (RNAO) (2002) [71]	Policy Statement: Racism	Policy Statement	RNAO is committed to an environment where all nurses and clients are treated with dignity and respect and where diversity is valued	Racism			Not applicable	RNAO is committed to achieving an environment where all members of the profession have equal opportunities to participate fully in the nursing profession to their maximum potential and where clients receive care that is respectful of cultural needs
Sands, S.et al. 2020 [77]	Caribbean nurse migration—a scoping review	Research – Scoping Review (5 stage framework)	The aim of this scoping review was to examine the amount, type, sources, distribution, and focus of the conceptual and empirical literature on migration of Caribbean nurses and to identify gaps in the literature	Migration of Caribbean nurses	National	Caribbean nurses	4/18 sources were Canadian (22%)	Findings include migration patterns and trends; post-migration experiences; past and present policies, programs, and practices; and consequences of migration to donor countries
Stewart, P. 2009 [66]	Themes of racial discrimination in the experience of black female nurse managers	Qualitative/ Interviews	This study examines the impact of race on the workplace experiences of Black women in nursing leadership positions	Nursing leadership	Ontario/ Toronto	African Canadian	Female 16 Caribbean nurse leaders	Racial discrimination in healthcare negatively impacted work experience for Black nurse managers
Villeneuve, M. 2003 [68]	Healthcare, Race and Diversity: Time to Act	Commentary	In Canadian nursing, beyond the staff nurse level there appear to be few persons of colour in formal decision-making, leadership or policy positions. When we look around at our nursing leaders, managers, directors, boards, faculty and decision-makers, we do not see the Canadian mosaic mirrored back. Rectifying these disparities across the health professions is long overdue, and the time has come to act	Diversity within Canadian healthcare	National	Visible minority, Black, African	N/A	In Canadian nursing, beyond the staff nurse level there appear to be few persons of colour in formal decision-making, leadership or policy positions

## Appendix 5

### Included studies (companion source)

- Boateng GO, Adams TL. “Drop dead … I need Your job”: An exploratory study of intra-professional conflict amongst nurses in Two, Ontario Cities. Soc Sci & Med. 2016;155:35–42.
- Etowa, J. Fostering healthy work environments for minority nurses in Nova Scotia. Nurs in Focus Fall. 2006;2:15–8.
- Etowa, J. Negotiating the boundaries of difference in the professional lives of black nurses. Int J Div in Organizations, Communities, and Nations. 2007;7(3):217–26.
- Etowa J, Sethi S, Thompson-Isherwood R. The substantive theory of surviving on the margin of a profession. Nurs Sci Quart. 2009;22(2):174–81.
- Flynn K. Race, class, and gender: Black nurses in Ontario, 1950–1980 [dissertation]. [Ottawa]: National Library of Canada/ Bibliothèque nationale du Canada; 2003.
- Flynn K. “I'm glad that someone is telling the nursing story.” J Black Stud. 2008;38(3):443–60.
- Flynn K. Beyond the Glass Wall: Black Canadian Nurses, 1940–1970. Nurs Hist Rev. 2009;17(1):129–52.
- Flynn, K. Moving beyond borders: A history of Black Canadian and Caribbean women in the diaspora. Toronto: University of Toronto Press; 2011.
- Flynn K. 'I'm not your typical nurse': Caribbean nurses in Britain and Canada. Women's History Magazine. 2012;69:26–32012.
- Flynn K. “She cannot be confined to her own region”: Nursing and nurses in the Caribbean, Canada, and the United Kingdom. Within and Without the Nation: Can Hist as Transnatl Hist. 2015;:228–50.
- Flynn K, Aladejebi F. Writing Black Canadian women’s history: Where we have been and where we are going. Read Can Women’s and Gend Hist. 2019;:63–89.
- Shkimba M, Flynn K. ʻIn England we did nursingʼ: Caribbean and British nurses in Great Britain and Canada, 1950–70. New Dir in Nurs Hist. 2004:157–73.
- Turrittin J, Hagey R, Guruge S, Collins E, Mitchell M. The experiences of professional nurses who have migrated to Canada: Cosmopolitan citizenship or democratic racism? Int J Nurs Stud. 2002;39(6):655–67.

## Data Availability

All data generated or analyzed during this study are included in this published article.
